# Supplementary material for: Sheared turbulent flows and wake dynamics of an idled floating tidal turbine
Source: Nat Commun. 2024 Sep 20;15:8244. doi: 10.1038/s41467-024-52578-x (PMC11415390; doi:10.1038/s41467-024-52578-x)
Supplement: Supplementary file 1 — Supplementary Information [file 41467_2024_52578_MOESM1_ESM.pdf]

Supplementary Information for:

Sheared turbulent flows and wake dynamics of an idled floating tidal turbine

Lilian Lieber[1,2*], Shaun Fraser[3], Daniel Coles[4], W. Alex M. Nimmo-Smith[2]

[1] Marine Biological Association of the United Kingdom, The Laboratory, Citadel Hill, Plymouth PL1 2PB, UK
[2] School of Biological and Marine Sciences, University of Plymouth, Plymouth PL4 8AA, UK
[3] UHI Shetland, Port Arthur, Scalloway, Shetland ZE1 0UN, UK
[4] School of Engineering, Computing and Mathematics, University of Plymouth, Plymouth PL4 8AA, UK

*Correspondence and requests for materials should be addressed to L.L. (email: lilian.lieber@mba.ac.uk)

Contents

Item	Page number(s)
**Fig. 1:** Overview of current velocities and physical scattering across the Fall of Warness from broad-scale transects during all tidal states	**2-15**
**Table 1:** Summary environmental information for each sampling transect for the fine-scale (red) and broad-scale (yellow) surveys	**16**
**Fig. 2**: Overview of additional ADCP inflow transect lines	**17-18**
**Fig. 3:** PIV-derived surface current magnitude and turbulence experienced by the O2 during ebb flow	**19**
**Fig. 4:** PIV-derived surface current magnitude and turbulence experienced by the O2 during flood flow	**20**
**Fig. 5:** Wake isolation and tracing using EK80 backscatter (Sv) from fine-scale transects	**21**
**Fig. 6:** Fine-scale transect extending 1600 m downstream of the O2 during ebb tidal flows	**22**
**Fig. 7:** PIV-derived surface current magnitude and turbulence across the O2 wake during ebb flow	**23**
**Fig. 8:** PIV-derived surface current magnitude and turbulence across the O2 wake during flood flow	**24**
**Fig. 9:** Survey vessel transect lines during fine-scale surveys	**25**
**Fig. 10:** Examples of backscattering sources and uncalibrated frequency response from bubbles and sediment	**26**
**Fig. 11:** Convergence of flow parameters derived from PIV data	**27**
**Supplementary Methods 1:** Large-scale Particle Image Velocimetry (LSPIV)	**27-28**
**Fig. 12:** Spatial (pixel space) distributions of cross-correlation at 25 randomly selected instantaneous vector locations upstream of the O2.	**28**
**Fig. 13:** Histograms of measured cross-correlation peak displacement to assess peak-locking	**29**
**Supplementary Methods 2:** Turbulence length scales	**29-39**
**Fig. 14:** Estimation of turbulence length scale (Lu).	**30**
**Fig. 15:** Comparison of the two methods for estimation of turbulence length scale (Lu).	**30**
**Supplementary Methods 3:** Assessment of Drone Stability and impact on turbulence parameters	**31**
**Table 2:** Drone stability parameters for hover segments and test flight over beach scene.	**31**
**Supplementary References**	**32**

Supplementary Figures and Tables

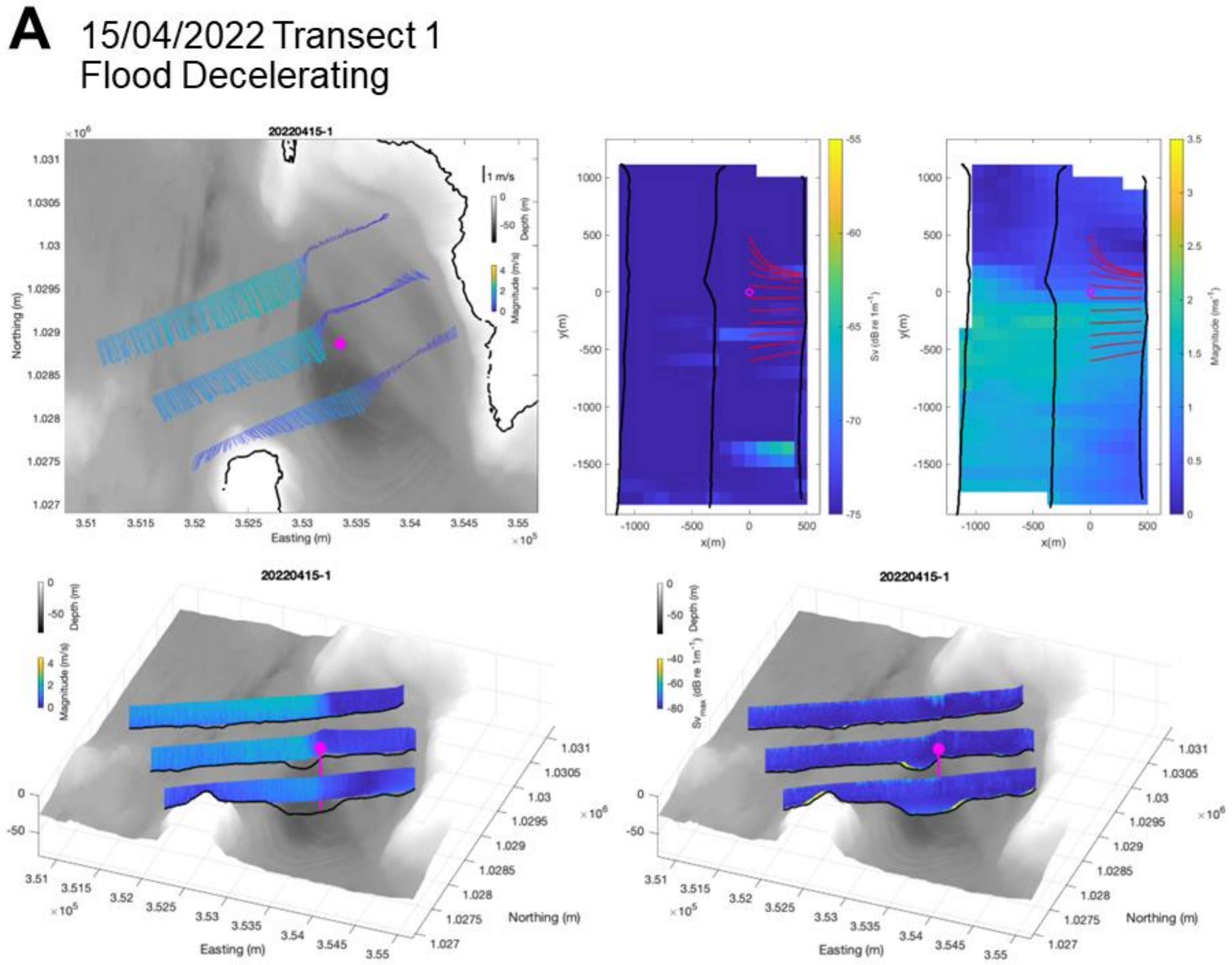

B 15/04/2022 Transect 2
Slack

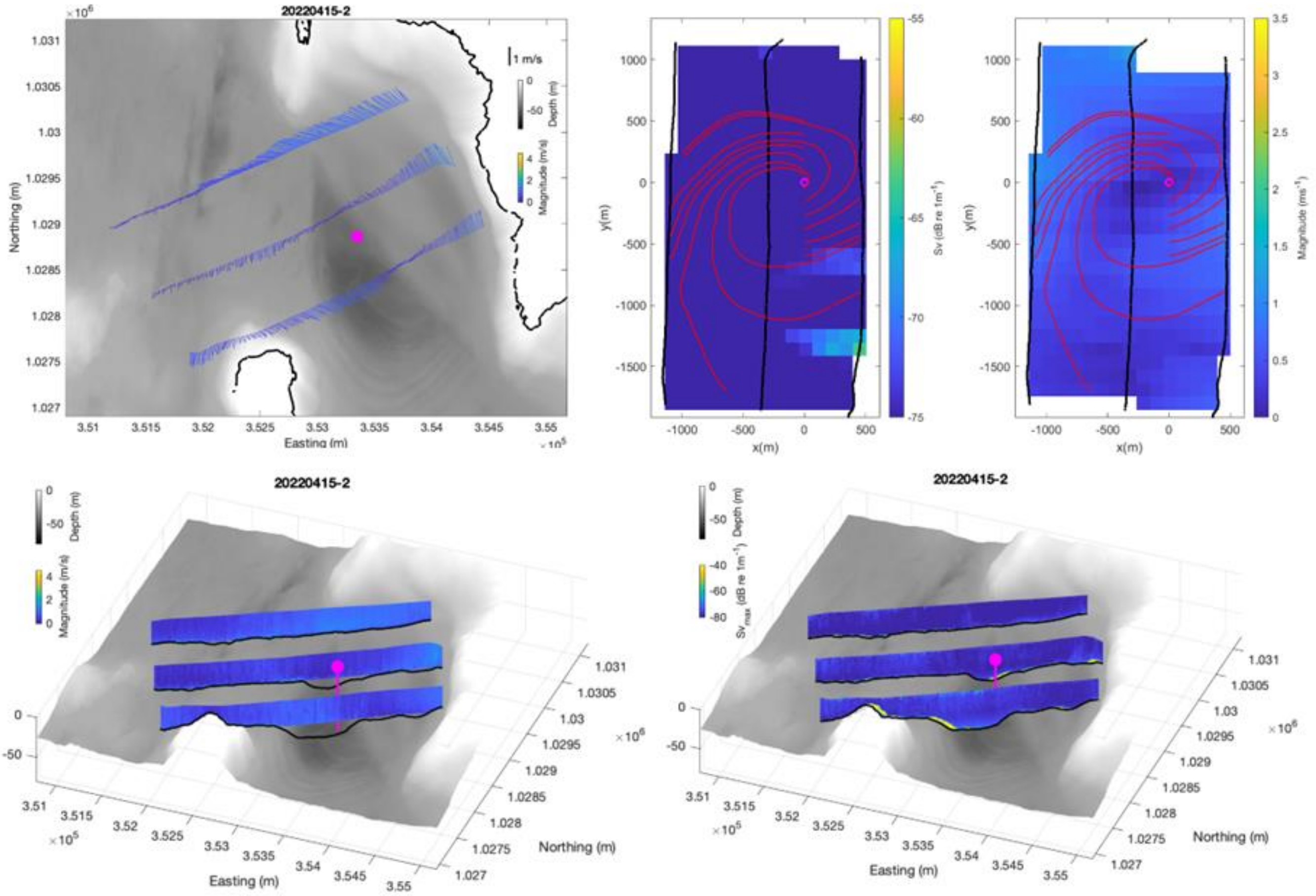

C 15/04/2022 Transect 3
Ebb Accelerating

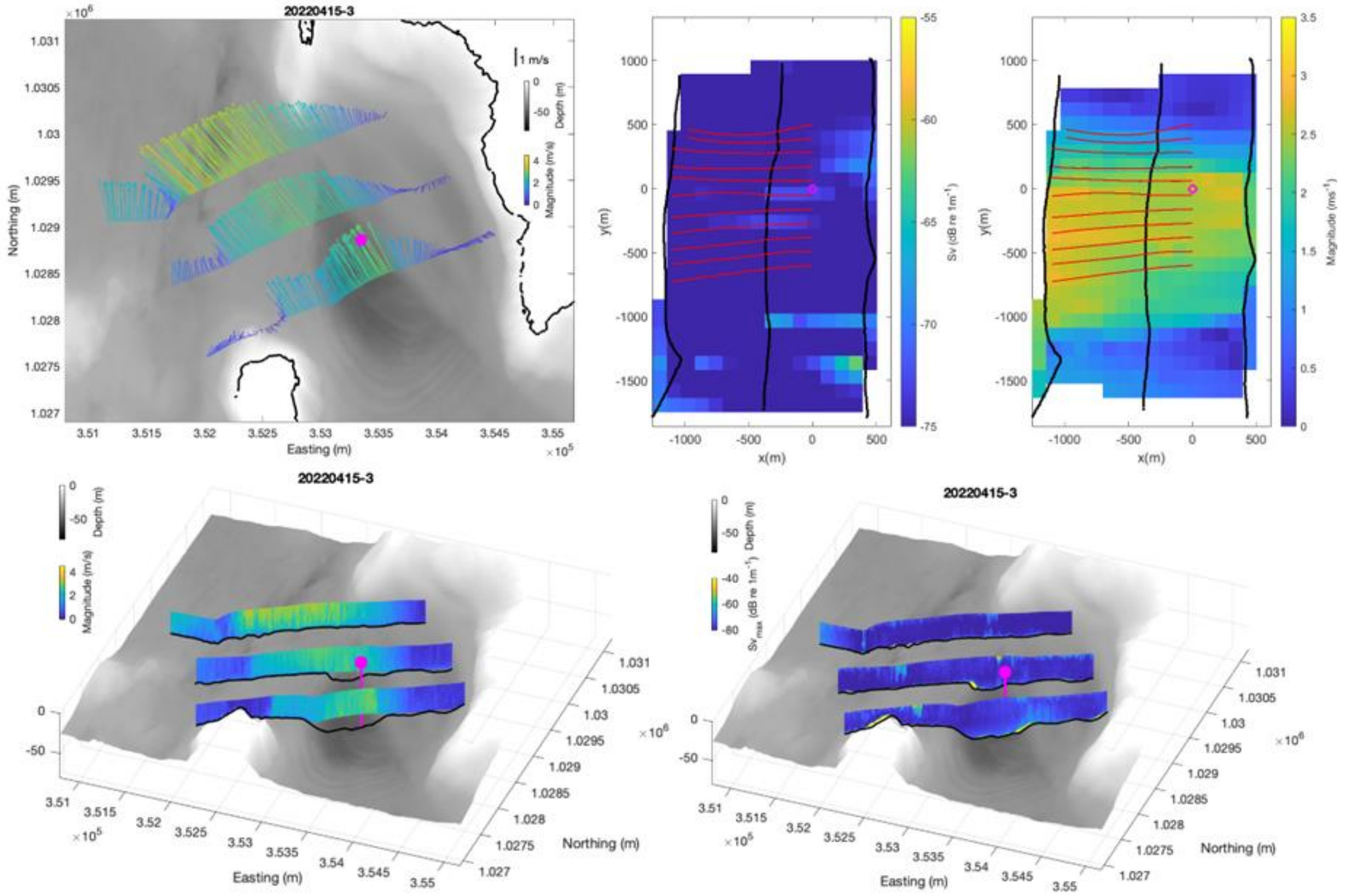

D 15/04/2022 Transect 4
Ebb Peak

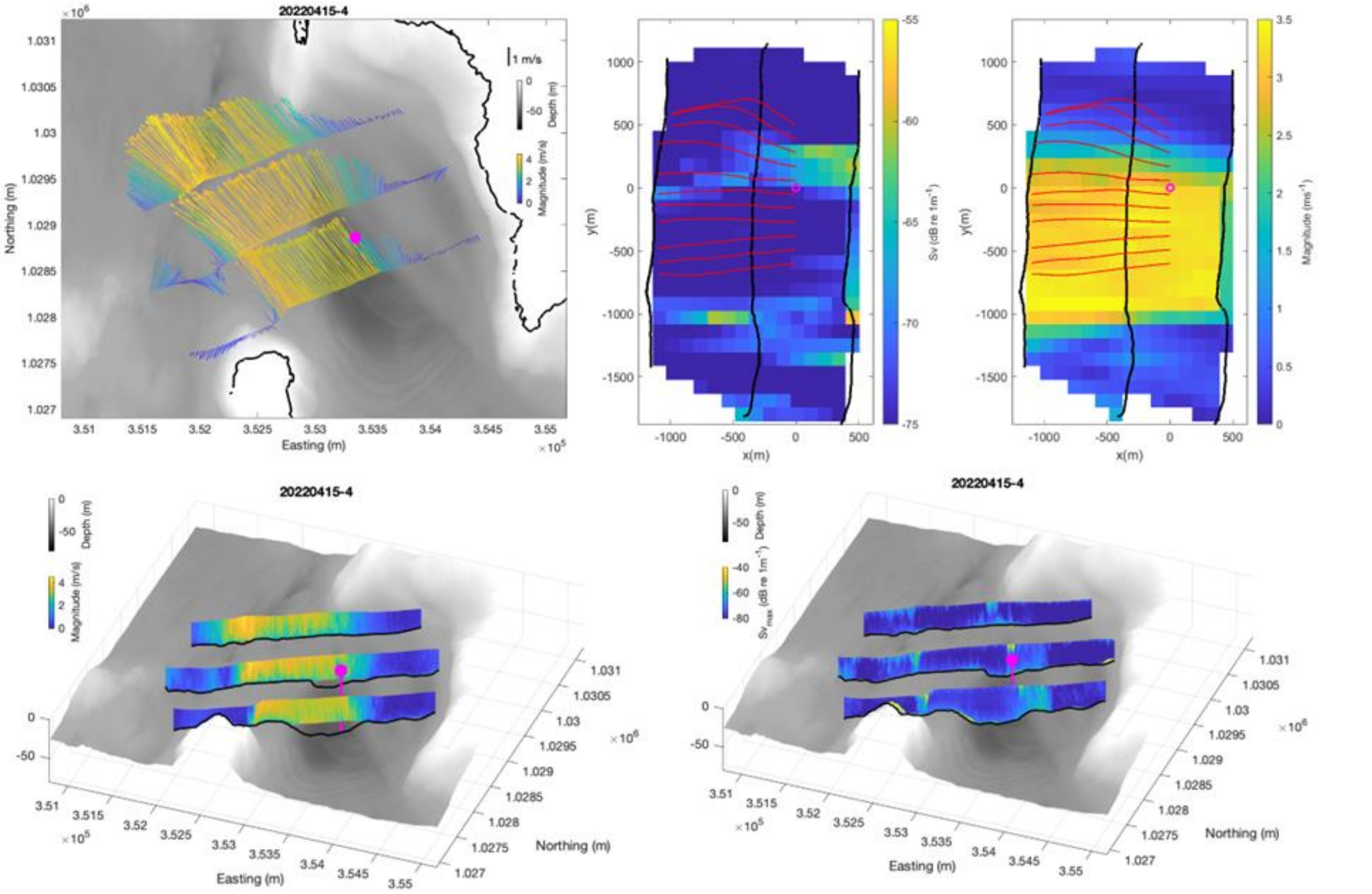

E 15/04/2022 Transect 5
Ebb Decelerating

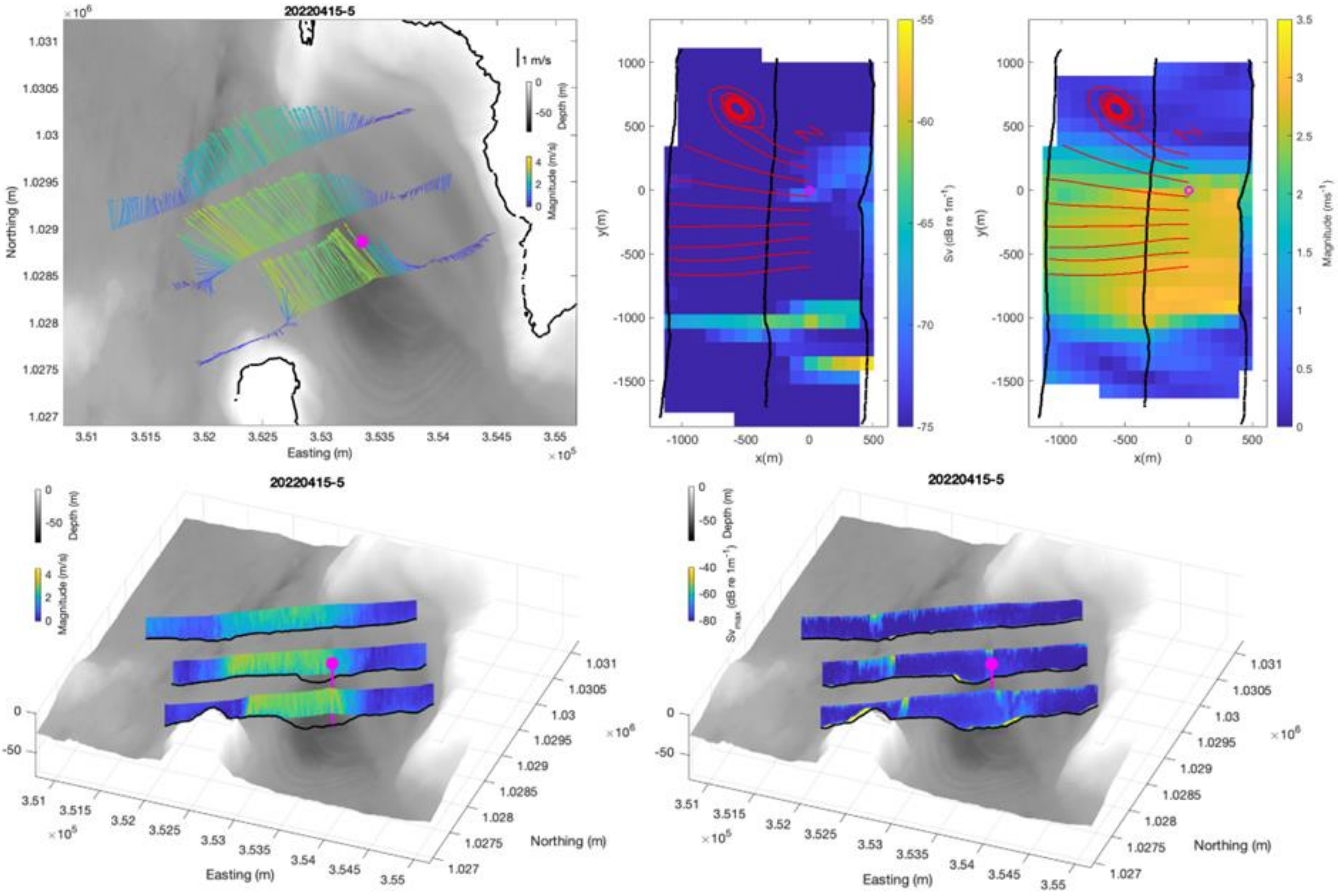

F 15/04/2022 Transect 6
Ebb Decelerating

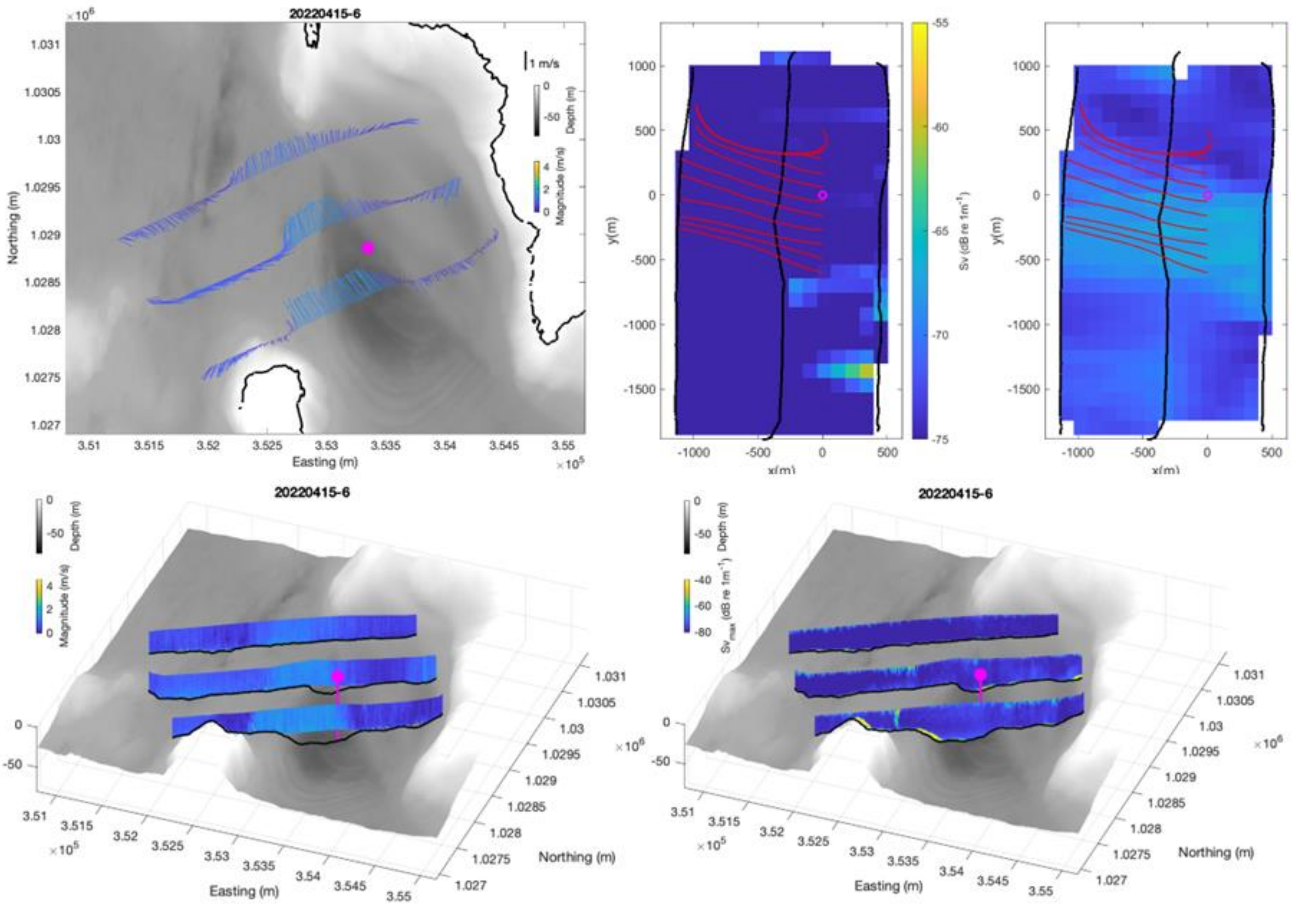

G 15/04/2022 Transect 7
Slack

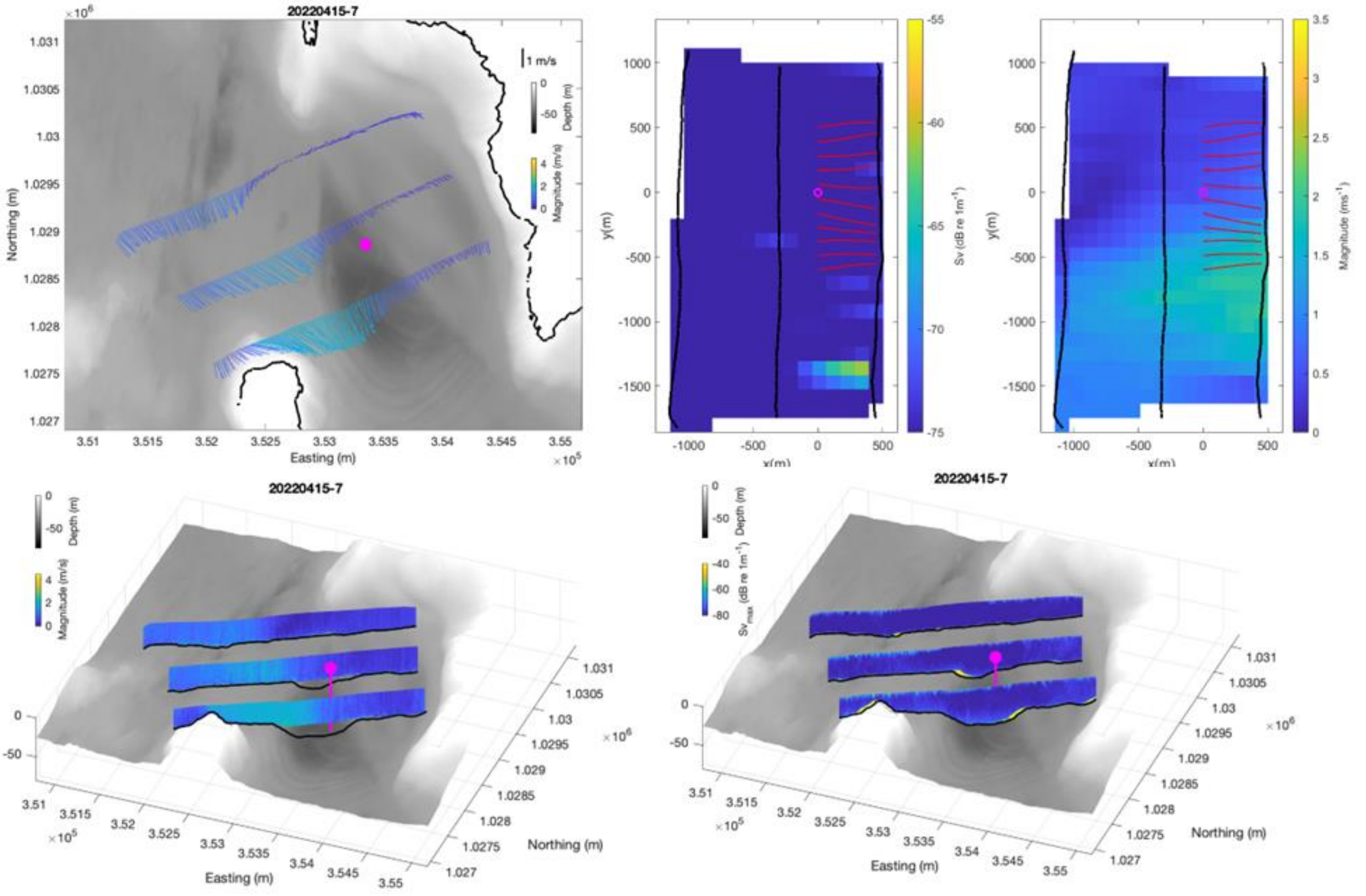

H 16/04/2022 Transect 1 Flood Decelerating

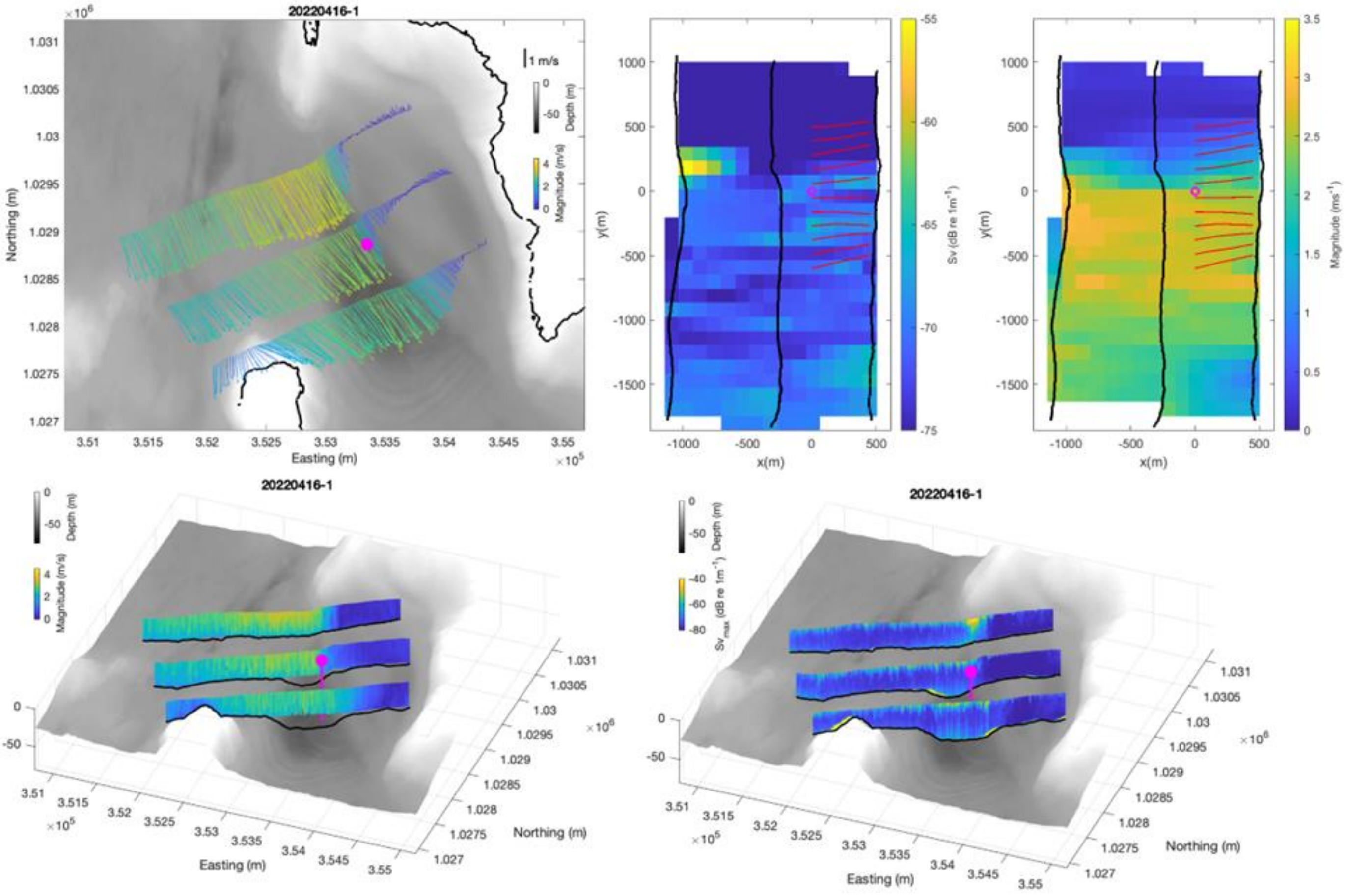

16/04/2022 Transect 2
Flood Decelerating

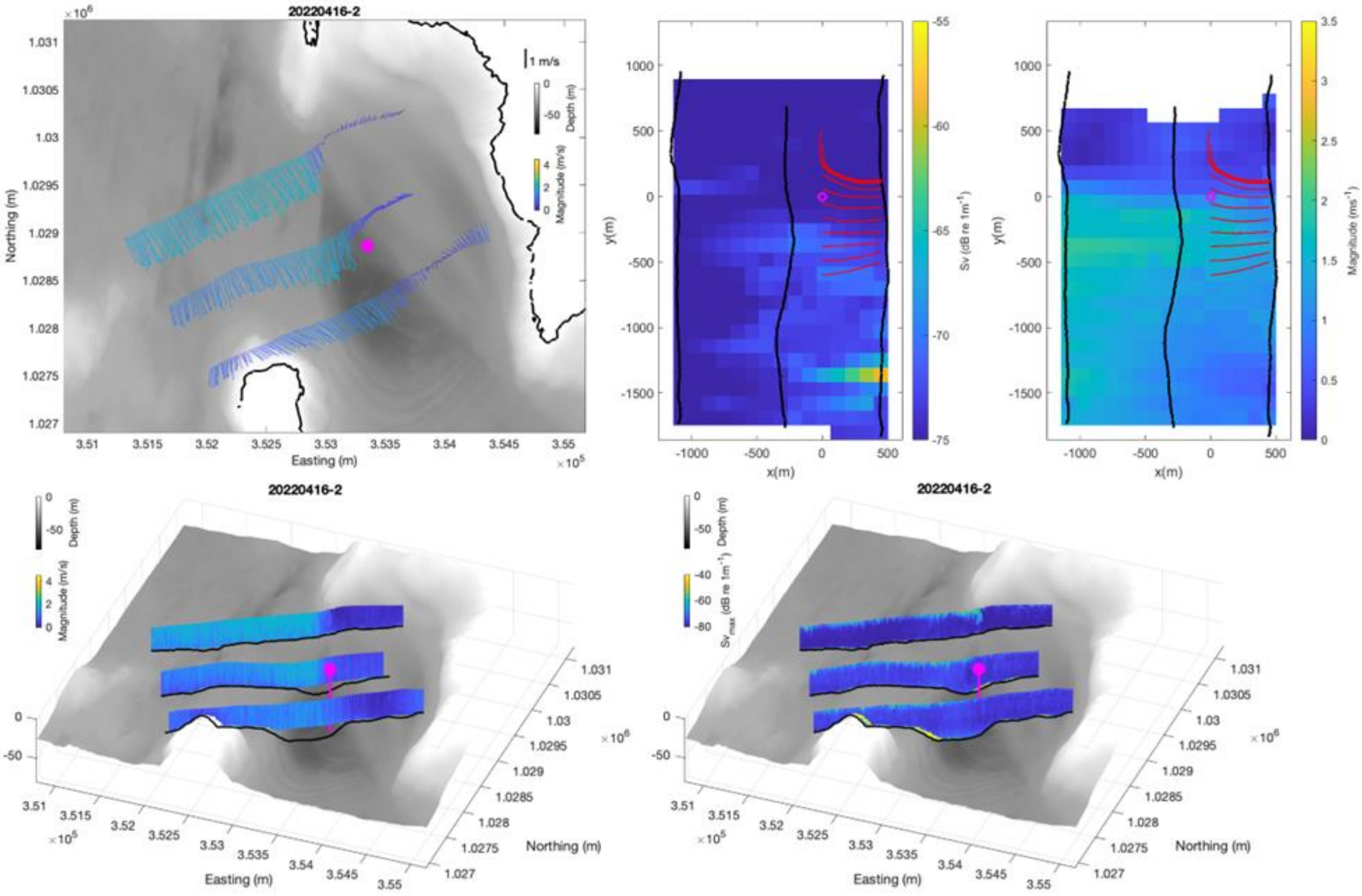

J 16/04/2022 Transect 3
Slack

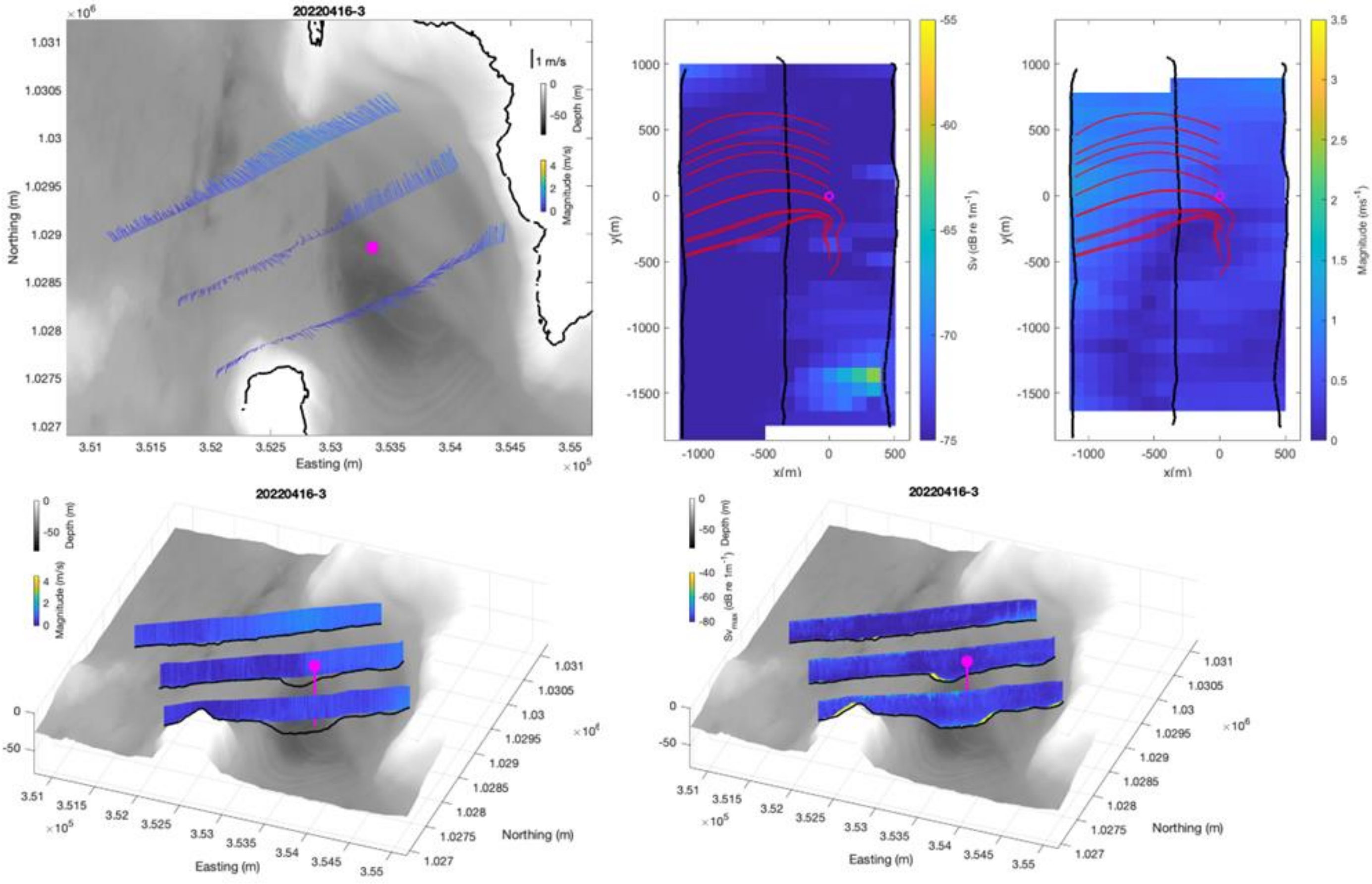

K 16/04/2022 Transect 4
Ebb Accelerating

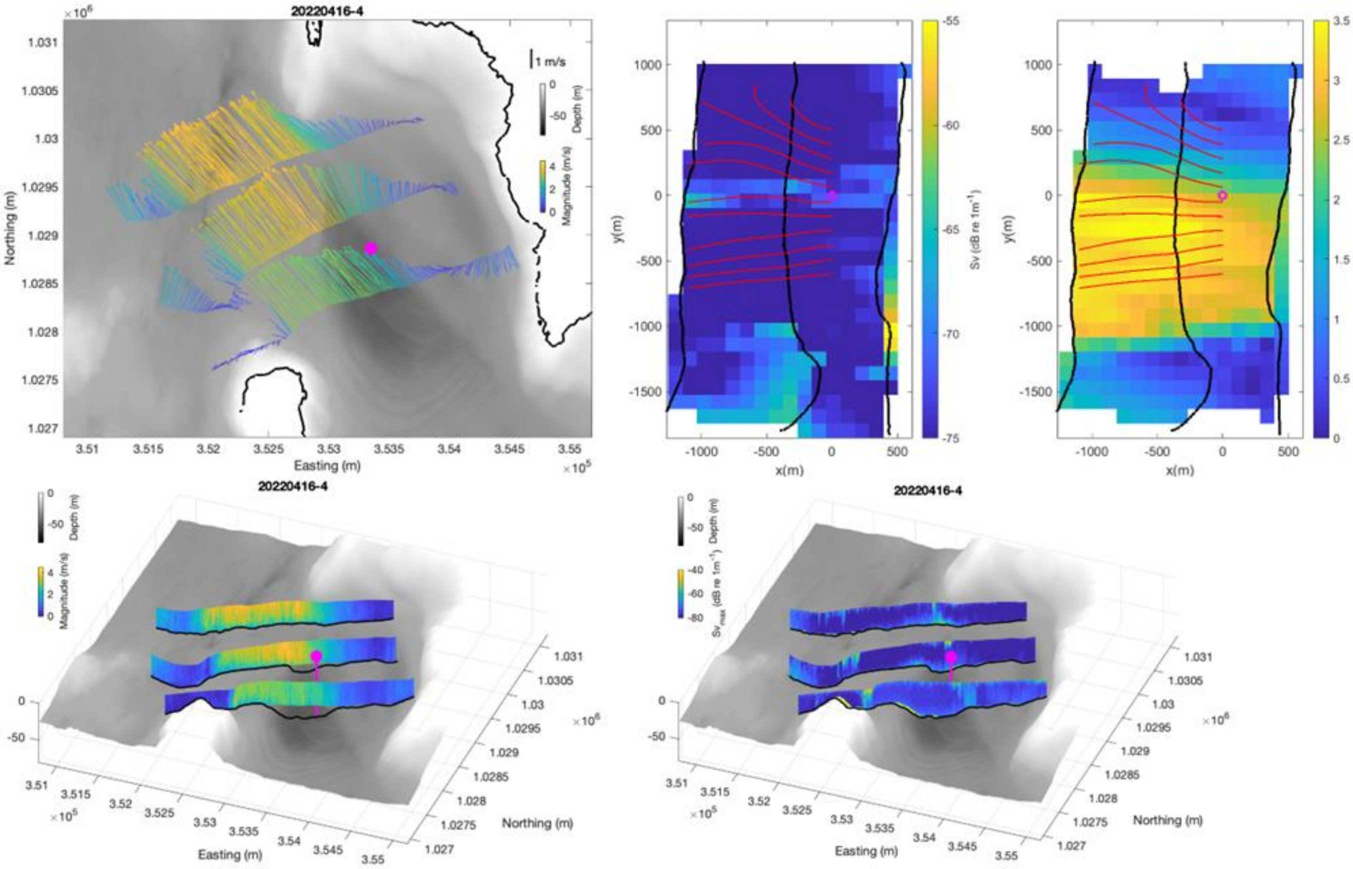

L 16/04/2022 Transect 5
Ebb Decelerating

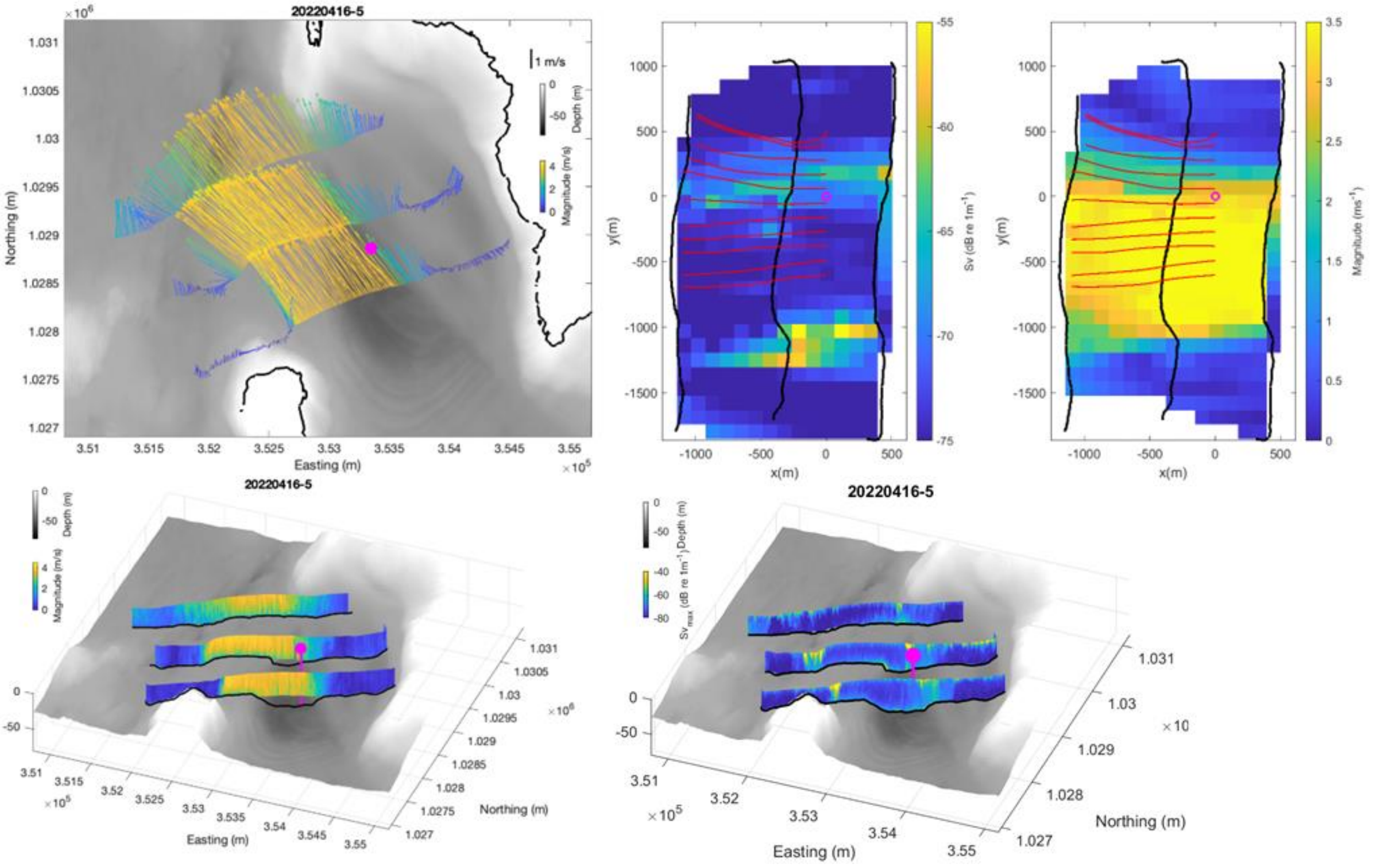

M 16/04/2022 Transect 6
Ebb Decelerating

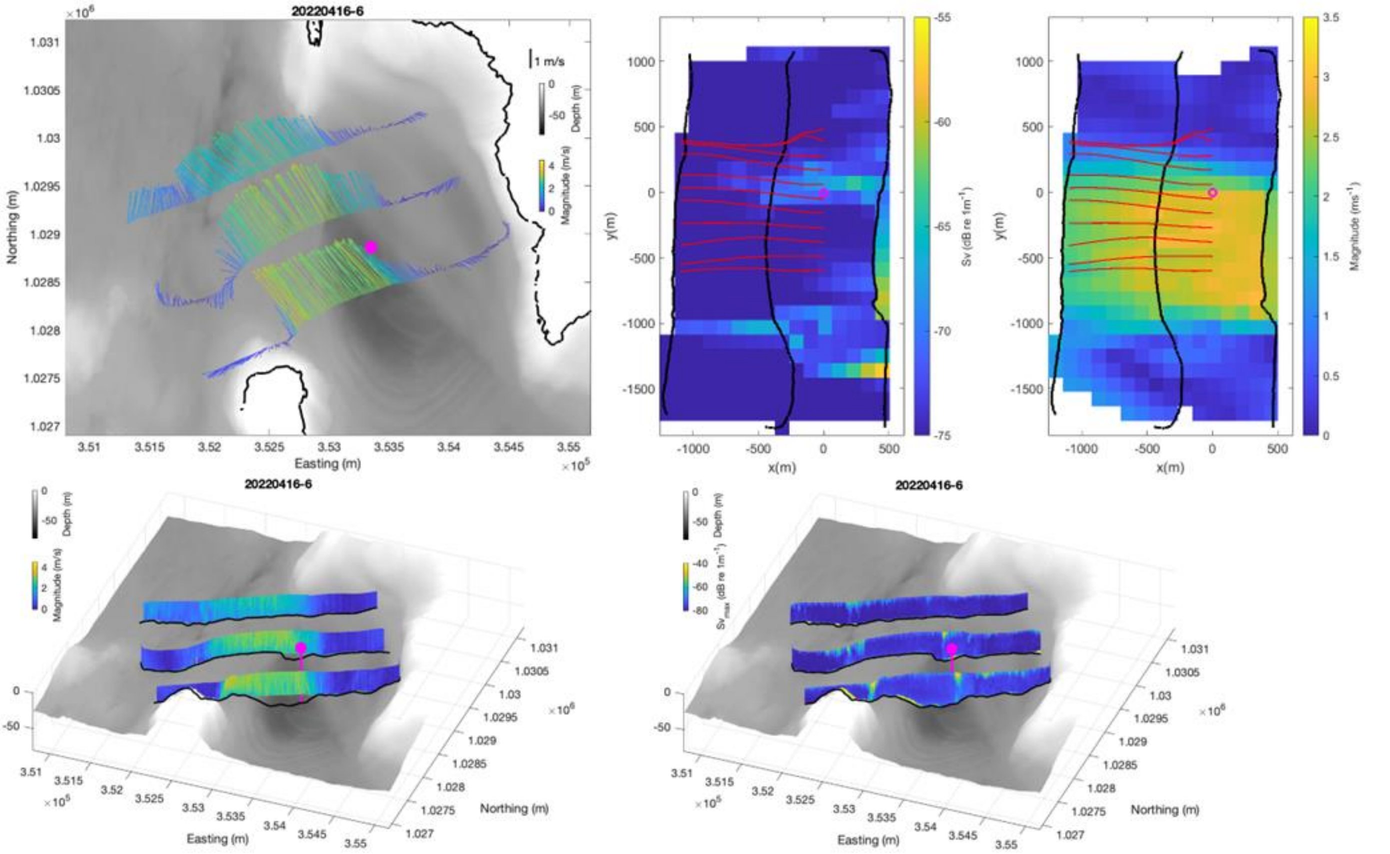

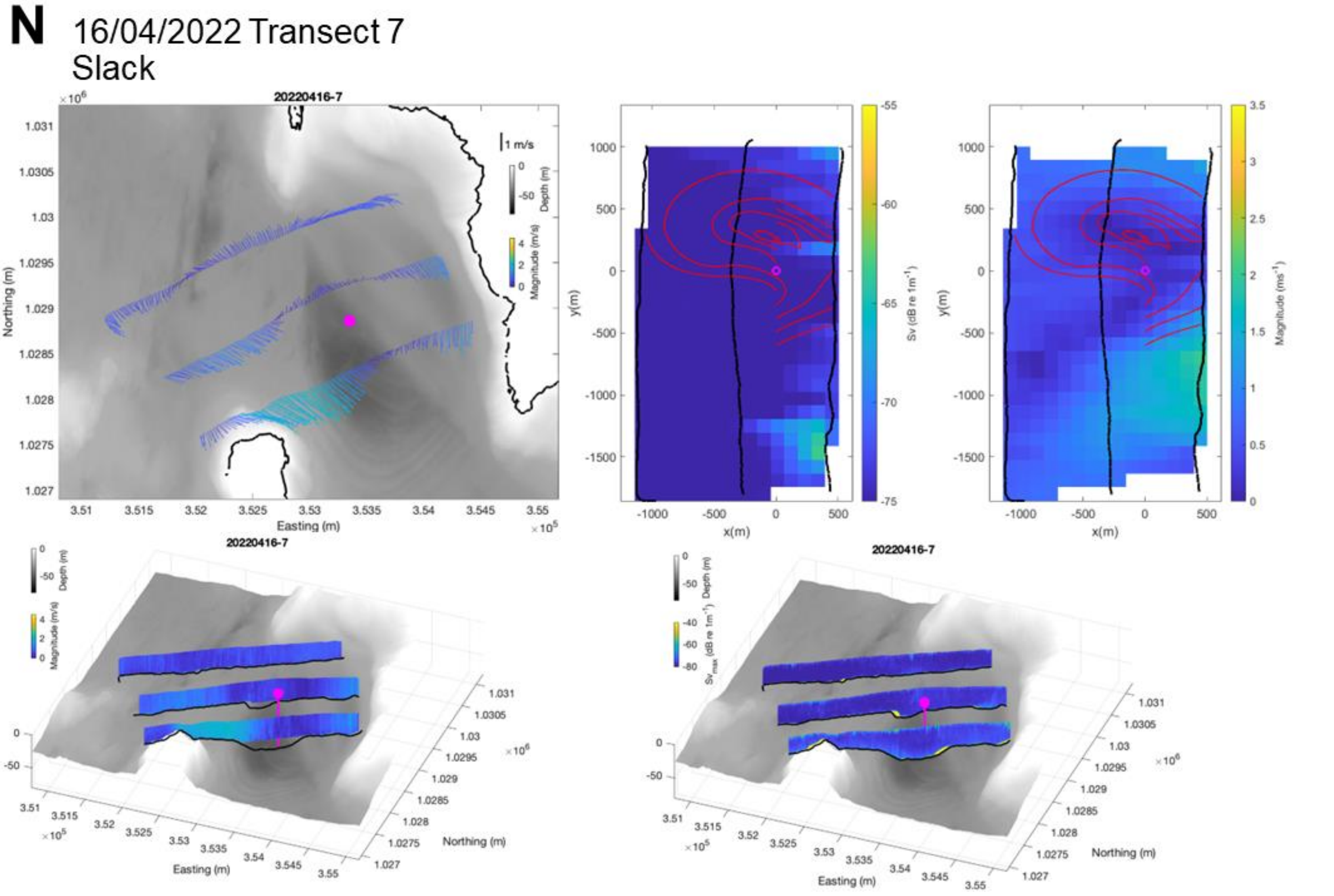

Fig. 1: Overview of current velocities and physical scattering across the Fall of Warness from broad-scale transects during all tidal states. Depth-averaged horizontal velocity (ms-^{1}) with vectors coloured by magnitude (upper left). Depth-averaged ADCP backscatter (S_v; dB re 1m^{-1}) and horizontal velocity magnitude data rotated to the local coordinate system and gridded at 5D where streamlines (red) show the behaviour of the flow cross- and downstream of the O2 (upper right). Vertical distributions of horizontal velocity magnitude (lower left) and ADCP-derived backscatter (lower right). For all plots, the mean location of the O2 tidal turbine is marked with a circle (magenta). Note, the O2 was not generating with the rotors left idle. Boundary shape files: Boundary-Line™ shape files downloaded from EDINA Digimap Ordnance Survey Service. Bathymetry:©Crown Copyright/ SeaZone Solutions Limited (2022). All Rights Reserved. Not to be used for navigation. Note, figures only provide an overview and the underlying ADCP-derived current flow data is available at DOI: https://doi.org/10.24382/244fae5d-2d16-4219-98f6-7aa96757ae49

Table 1: Summary environmental information for each sampling transect for the fine-scale (red) and broad-scale (yellow) surveys. Mean velocity is the depth-mean velocity extracted from the Orbital ADCPs, averaged over the transect period, with associated standard deviation (SD). Acc denotes the acceleration in depth mean velocity from the Orbital ADCPs calculated using a linear regression over the transect period. Mean velocity and acceleration determine the Tidal State with Peak indicating strongest flows. Wind speed and direction, Wave height and Mean heading of the O2, and displacement were all measured by sensors on the platform. Wave heights less than 0.5 m should be treated with caution. Drone in-flight mean wind speed was extracted from the Airdata UAV log management system.

Date	Transect Number	Transect start time (GMT)	Transect end time (GMT)	Mean velocity (ms^{-1}) ± SD	Acc (ms^{-1} hr^{-1})	Tidal State (Accelerating, Decelerating, Slack, Peak flows)	Mean Wind Speed (ms^{-1})	Mean Wind Dir (°)	Drone take-off time (GMT) for hovers	Drone landing time (GMT) for hovers	Drone in-flight mean wind speed (ms^{-1}) at 60 m altitude ± SD	Mean Wave Height (m) ± SD	Mean heading of O2 (°) ± SD	Streamwise displaceme nt of O2 (m) ± SD	Cross-stream displaceme nt of O2 (m) ± SD
13/04/2022	1	10:46	11:16	-1.80 ± 0.10	0.57	Ebb Acc	6.57	7.0	-	-	-	1.7 ± 0.2	334.8 ± 0.9	-7.6 ± 0.6	2.1 ± 1.4
13/04/2022	2	12:04	12:45	-2.31 ± 0.06	0.17	Ebb Acc	9.84	3.3	-	-	-	0.7 ± 0.1	329.0 ± 1.4	-12.2 ± 0.4	-6.9 ± 1.5
13/04/2022	3	13:10	13:43	-2.16 ± 0.08	-0.35	Ebb Decel	2.81	12.8	-	-	-	0.5 ± 0.0	329.0 ± 1.9	-11.6 ± 0.7	-7.3 ± 2.7
13/04/2022	4	14:00	14:28	-1.56 ± 0.24	-0.81	Ebb Decel	8.50	7.6	-	-	-	0.4 ± 0.0	329.9 ± 1.4	-7.8 ± 1.2	-9.9 ± 4.1
13/04/2022	5	15:14	15:39	-0.50 ± 0.17	-1.30	Slack	12.91	12.4	-	-	-	0.6 ± 0.0	334.2 ± 0.8	-0.2 ± 0.2	-2.3 ± 2.3
14/04/2022	1	07:53	08:25	-	-	-	5.60	108.7	07:35	07:48	5.34 ± 0.66	-	-	-	-
14/04/2022	2	09:02	09:24	-	-	-	11.60	118.8	08:46	08:59	4.78 ± 0.62	-	-	-	-
14/04/2022	3	10:35	10:56	-0.96 ± 0.34	2.96	Ebb Acc	15.23	132.5	10:15	10:28	6.85 ± 0.83		332.0 ± 2.1	-2.9 ± 3.0	-2.0 ± 8.6
14/04/2022	4	11:40	12:25	-2.55 ± 0.35	1.49	Ebb Acc	7.72	100.4	11:20	11:35	6.55 ± 0.64	0.3 ± 0.0	329.7 ± 2.7	-13.3 ± 1.8	-6.0 ± 3.2
14/04/2022	5	13:43	14:09	-2.57 ± 0.05	-0.11	Ebb Decel	-	-	13:18	13:33	7.12 ± 0.44	0.3 ± 0.0	328.9 ± 1.9	-14.0 ± 0.4	-7.1 ± 2.0
14/04/2022	6	15:15	15:33	-1.31 ± 0.17	-1.60	Ebb Decel	-	-	-	-	-	0.3 ± 0.0	333.0 ± 1.0	-5.8 ± 0.9	-8.7 ± 2.6
14/04/2022	7	16:16	16:33	-0.19 ± 0.06	0.45	Slack	-	-	15:56	16:11	8.20 ± 0.49	0.4 ± 0.0	335.4 ± 0.2	-0.3 ± 0.2	-2.7 ± 0.8
15/04/2022	1	08:50	09:48	1.64 ± 0.07	-1.49	Flood Decel	-	-	-	-	-	0.3 ± 0.1	330.5 ± 3.0	6.1 ± 2.8	1.1 ± 3.5
15/04/2022	2	10:03	10:50	-0.09 ± 0.14	0.37	Slack	9.87	129.1	-	-	-	0.2 ± 0.0	331.7 ± 1.8	2.5 ± 0.2	1.5 ± 3.8
15/04/2022	3	11:30	12:28	-2.58 ± 0.25	0.39	Ebb Acc	14.11	138.9	-	-	-	0.3 ± 0.0	332.1 ± 2.9	-13.2 ± 1.8	-3.2 ± 3.9
15/04/2022	4	12:52	13:52	-3.24 ± 0.11	0.00	Ebb Peak	17.04	143.1	-	-	-	0.4 ± 0.0	329.3 ± 1.8	-17.0 ± 0.7	-5.8 ± 1.7
15/04/2022	5	14:30	15:22	-2.59 ± 0.25	-0.90	Ebb Decel	18.90	143.2	-	-	-	0.4 ± 0.0	329.3 ± 2.3	-13.8 ± 1.4	-7.5 ± 3.3
15/04/2022	6	15:59	16:42	-0.78 ± 0.20	-0.93	Ebb Decel	15.27	143.0	-	-	-	0.4 ± 0.0	334.0 ± 0.9	-2.1 ± 1.5	-4.7 ± 4.6
15/04/2022	7	16:59	17:41	0.37 ± 0.50	2.01	Slack	8.60	139.7	-	-	-	0.5 ± 0.0	333.3 ± 2.3	3.6 ± 2.1	4.0 ± 6.1
16/04/2022	1	08:08	09:02	2.72 ± 0.26	-0.88	Flood Decel	16.12	168.1	-	-	-	0.6 ± 0.1	325.1 ± 2.0	16.3 ± 1.1	6.1 ± 2.1
16/04/2022	2	09:40	10:26	0.94 ± 0.41	-1.74	Flood Decel	18.02	164.5	-	-	-	0.8 ± 0.2	330.8 ± 3.5	6.2 ± 2.8	0.8 ± 4.8
16/04/2022	3	10:48	11:35	-0.46 ± 0.40	1.65	Slack	13.10	161.0	-	-	-	1.5 ± 0.3	329.5 ± 1.7	2.5 ± 0.6	8.2 ± 5.5
16/04/2022	4	12:20	13:15	-2.88 ± 0.30	1.03	Ebb Acc	18.63	166.5	-	-	-	2.0 ± 0.3	330.3 ± 3.1	-14.9 ± 1.5	-4.5 ± 3.0
16/04/2022	5	13:50	14:51	-3.36 ± 0.09	-0.24	Ebb Decel	23.06	172.9	-	-	-	0.8 ± 0.1	328.8 ± 2.1	-17.1 ± 0.6	-6.7 ± 1.9
16/04/2022	6	15:20	16:11	-2.45 ± 0.29	-1.13	Ebb Decel	27.48	172.9	-	-	-	0.6 ± 0.1	328.3 ± 1.7	-13.3 ± 1.5	-7.9 ± 1.9
16/04/2022	7	17:09	17:53	-0.23 ± 0.22	-0.61	Slack	24.53	154.7	-	-	-	0.7 ± 0.1	335.3 ± 0.5	0.3 ± 0.5	-1.9 ± 1.2
17/04/2022	1	08:21	08:42	3.18 ± 0.22	1.25	Flood Acc	2.56	111.1	07:52	08:06	3.55 ± 0.85	0.4 ± 0.0	324.5 ± 1.8	18.1 ± 0.8	7.0 ± 1.7
17/04/2022	2	09:24	09:43	2.54 ± 0.11	0.27	Flood Acc	1.73	85.7	09:05	09:19	4.22 ± 0.16	0.5 ± 0.0	325.5 ± 2.8	15.2 ± 0.7	5.4 ± 2.7
17/04/2022	3	10:22	10:43	1.10 ± 0.40	-3.77	Flood Decel	5.25	125.2	10:03	10:16	5.35 ± 0.40	0.4 ± 0.0	330.0 ± 3.4	6.3 ± 2.8	1.0 ± 5.2
17/04/2022	4	11:36	11:56	-0.28 ± 0.11	0.87	Slack	9.43	139.6	11:18	11:29	10.70 ± 0.45	0.7 ± 0.0	329.6 ± 1.2	2.2 ± 0.3	6.9 ± 3.5
17/04/2022	5	12:43	13:09	-2.82 ± 0.10	0.51	Ebb Acc	8.47	128.7	12:23	12:36	7.69 ± 0.62	0.9 ± 0.0	330.8 ± 4.8	-14.5 ± 1.0	-4.6 ± 4.8
17/04/2022	6	13:45	14:01	-3.18 ± 0.06	-0.19	Ebb Decel	7.21	117.7	13:26	13:41	8.00 ± 0.36	-	329.2 ± 2.1	-16.1 ± 0.4	-5.5 ± 1.9

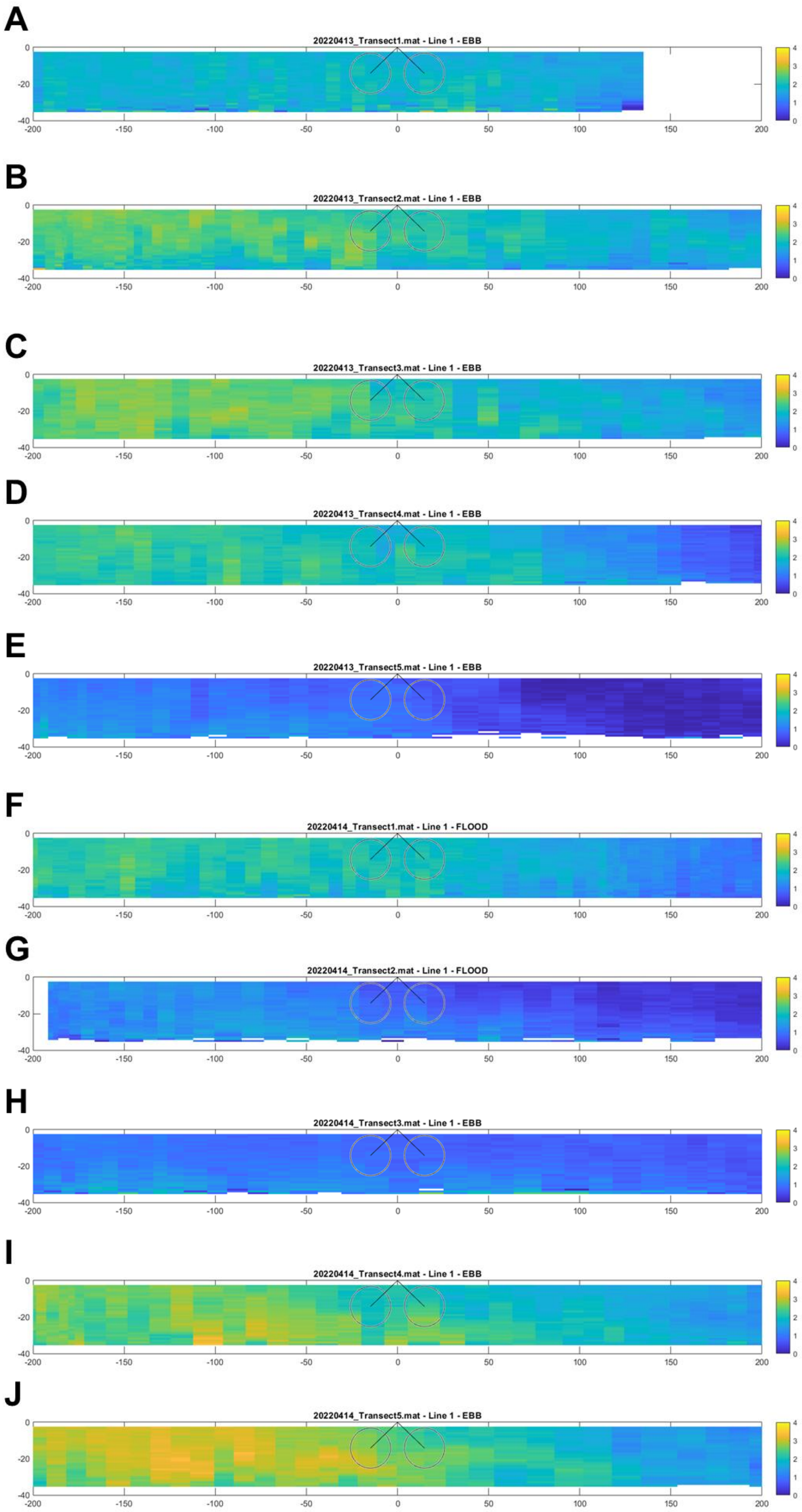
A
20220413_Transect1.mat - Line 1 - EBB
B
20220413_Transect2.mat - Line 1 - EBB
C
20220413_Transect3.mat - Line 1 - EBB
D
20220413_Transect4.mat - Line 1 - EBB
E
20220413_Transect5.mat - Line 1 - EBB
F
20220414_Transect1.mat - Line 1 - FLOOD
G
20220414_Transect2.mat - Line 1 - FLOOD
H
20220414_Transect3.mat - Line 1 - EBB
I
20220414_Transect4.mat - Line 1 - EBB
J
20220414_Transect5.mat - Line 1 - EBB

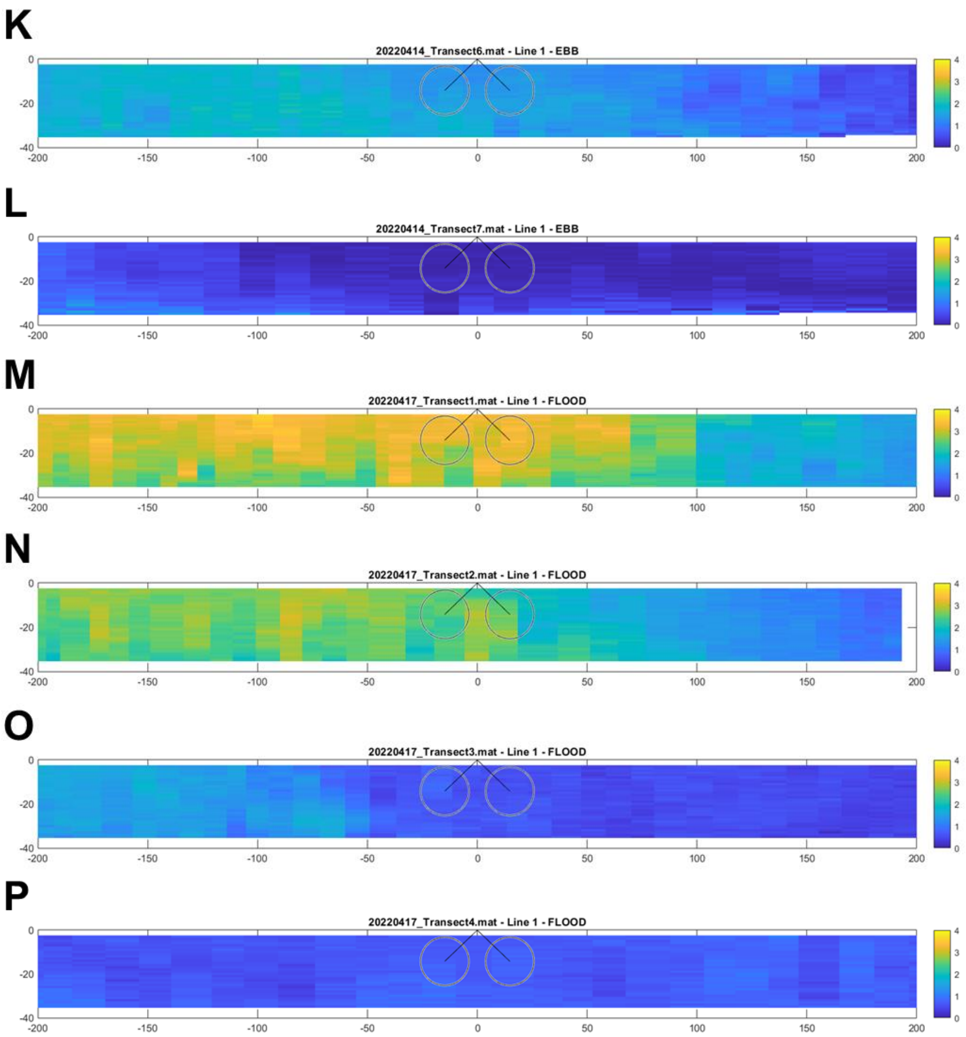

Fig. 2: Overview of additional ADCP inflow transect lines. Transects showing absolute streamwise horizontal velocity (|U| ms^{-1}) upstream (100 m or 5D) of the O2. For reference, the downstream location of the O2 is superimposed (hull structure, rotor arms and rotor-swept area). Note, figures only provide an overview and the underlying ADCP-derived current flow data (including the entire transect rather than just the inflow line) is available at DOI: https://doi.org/10.24382/244fae5d-2d16-4219-98f6-7aa96757ae49

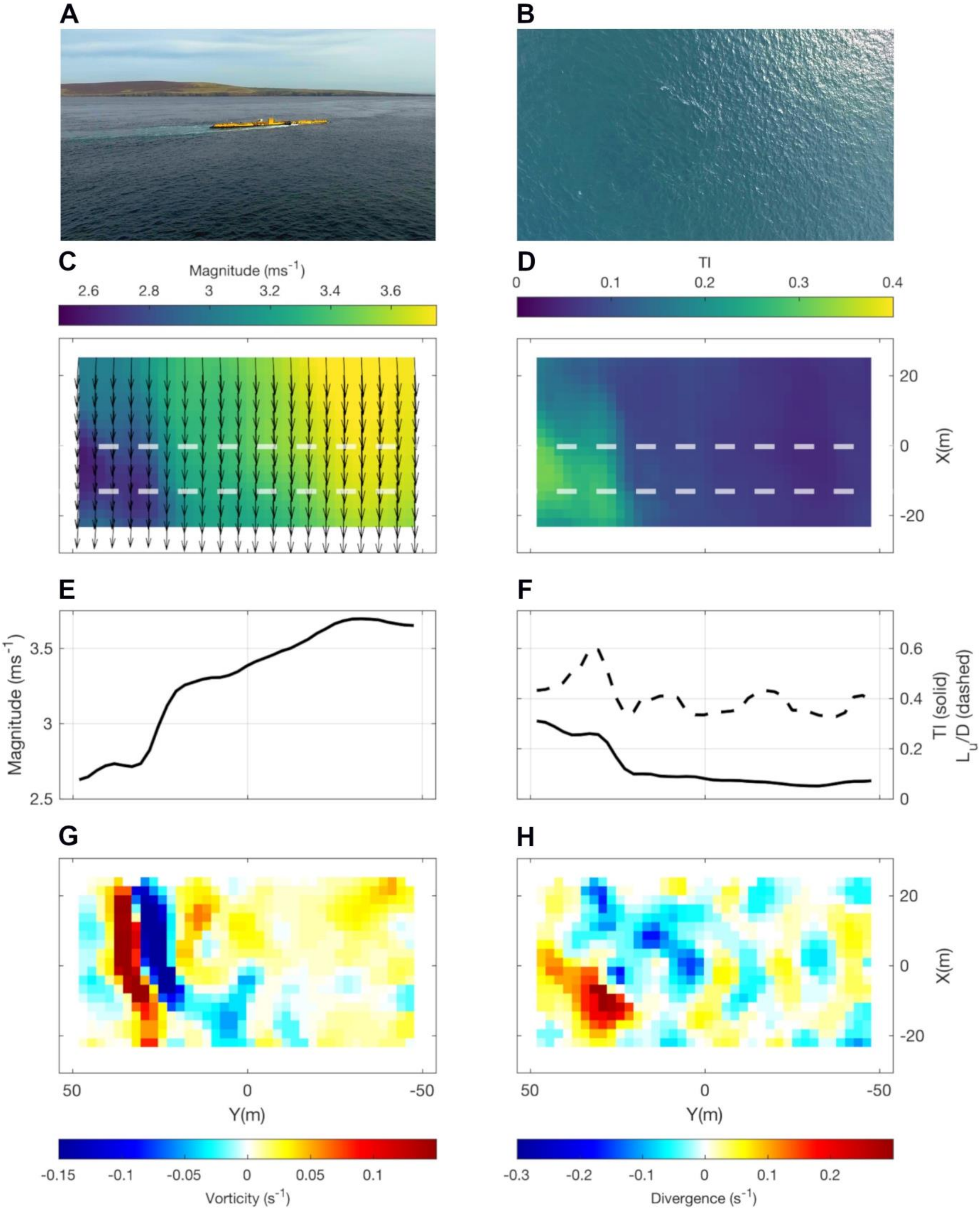

Fig. 3: PIV-derived surface current magnitude and turbulence experienced by the O2 during ebb flow. A Oblique aerial drone image approaching the O2 platform during ebb flow (17/04/2022, mean ebb velocity = 3.18 ms^{-1}). **B** Aerial image of drone hover field of view (T6, hover 1, altitude=65 m) upstream (5.5D) of the O2, with boils visible on the left side (towards Eday). **C** Mean flow field coloured by horizontal velocity magnitude with velocity vectors overlaid and (**D**), turbulence intensity (TI), as calculated from the 2-min hover. **E** Spatial and temporal mean horizontal velocity magnitude and (**F**), turbulence intensity both calculated across the region bounded by dashed, white lines in **C** and **D**, highlighting the difference in horizontal velocity magnitude and TI on either side of the platform. The dashed lines in **F** are the turbulence length scales $L_u(\Delta X)$ normalised by the rotor diameter D. Regions of (**G**) vorticity (positive = anti-clockwise) and (**H**), divergence (positive = upwelling) and convergence (negative = downwelling) on either side of the O2. The local coordinate system is centred around the mean O2 location.

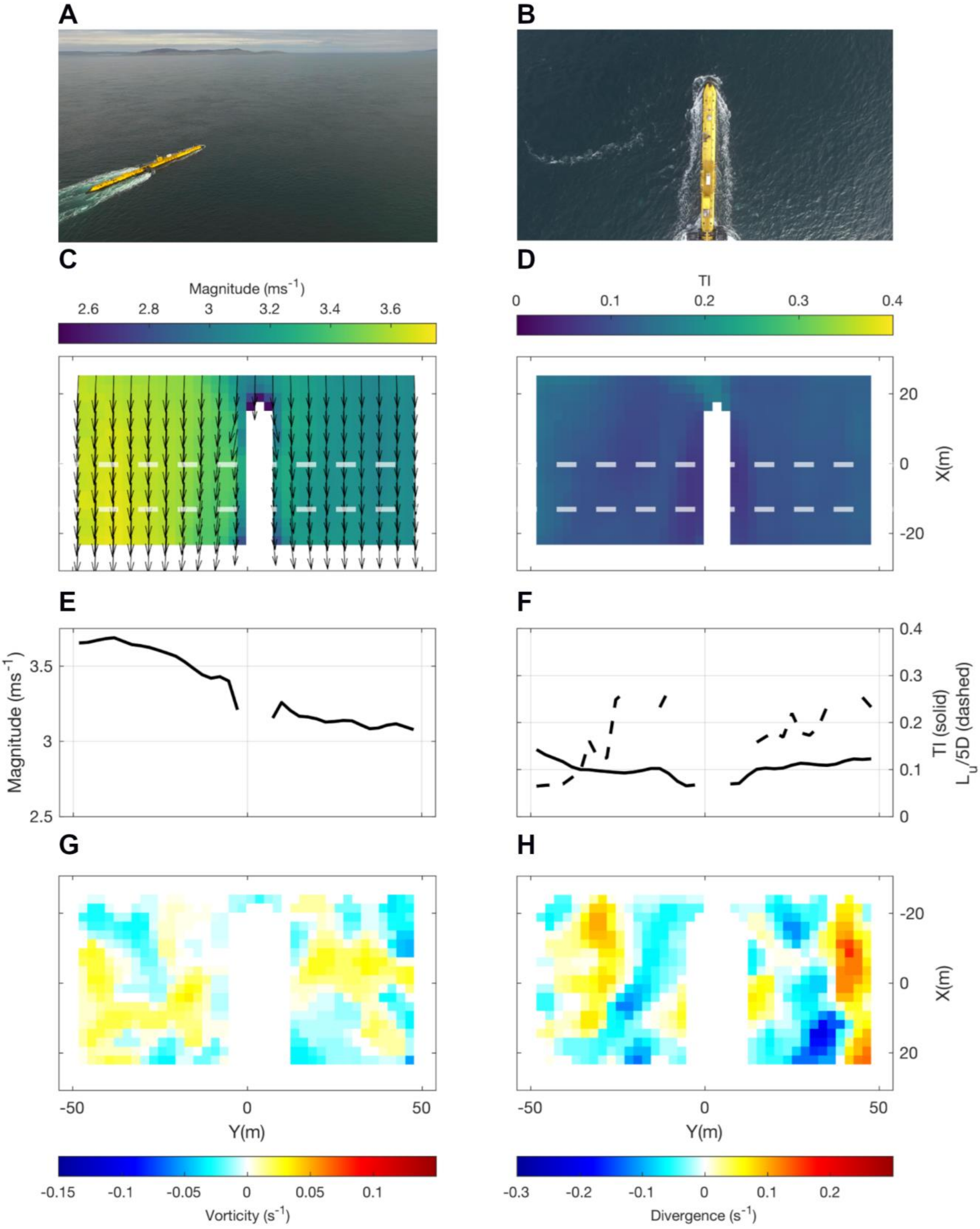

Fig. 4: PIV-derived surface current magnitude and turbulence experienced by the O2 during flood flow. A Oblique aerial drone image approaching the O2 platform during flood flow (17/04/2022, flood velocity = 3.2 ms^{-1}). **B** Aerial image of drone hover field of view (T1, hover 2, altitude=65 m) over the O2. **C** Mean flow field coloured by horizontal velocity magnitude with velocity vectors overlaid and (**D**), turbulence intensity (TI), as calculated from the 2-min hover. Note, the O2 has been masked. **E** Spatial and temporal mean horizontal velocity magnitude and (**F**), turbulence intensity both calculated across the region bounded by dashed, white lines in **C** and **D**, highlighting the difference in horizontal velocity magnitude and TI on either side of the platform. The dashed lines in **F** are the turbulence length scales $L_u(\Delta X)$ normalised by the rotor diameter D. Regions of (**G**) vorticity (positive = anti-clockwise) and (**H**), divergence (positive = upwelling) and convergence (negative = downwelling) on either side of the O2. The local coordinate system is centred around the mean O2 location.

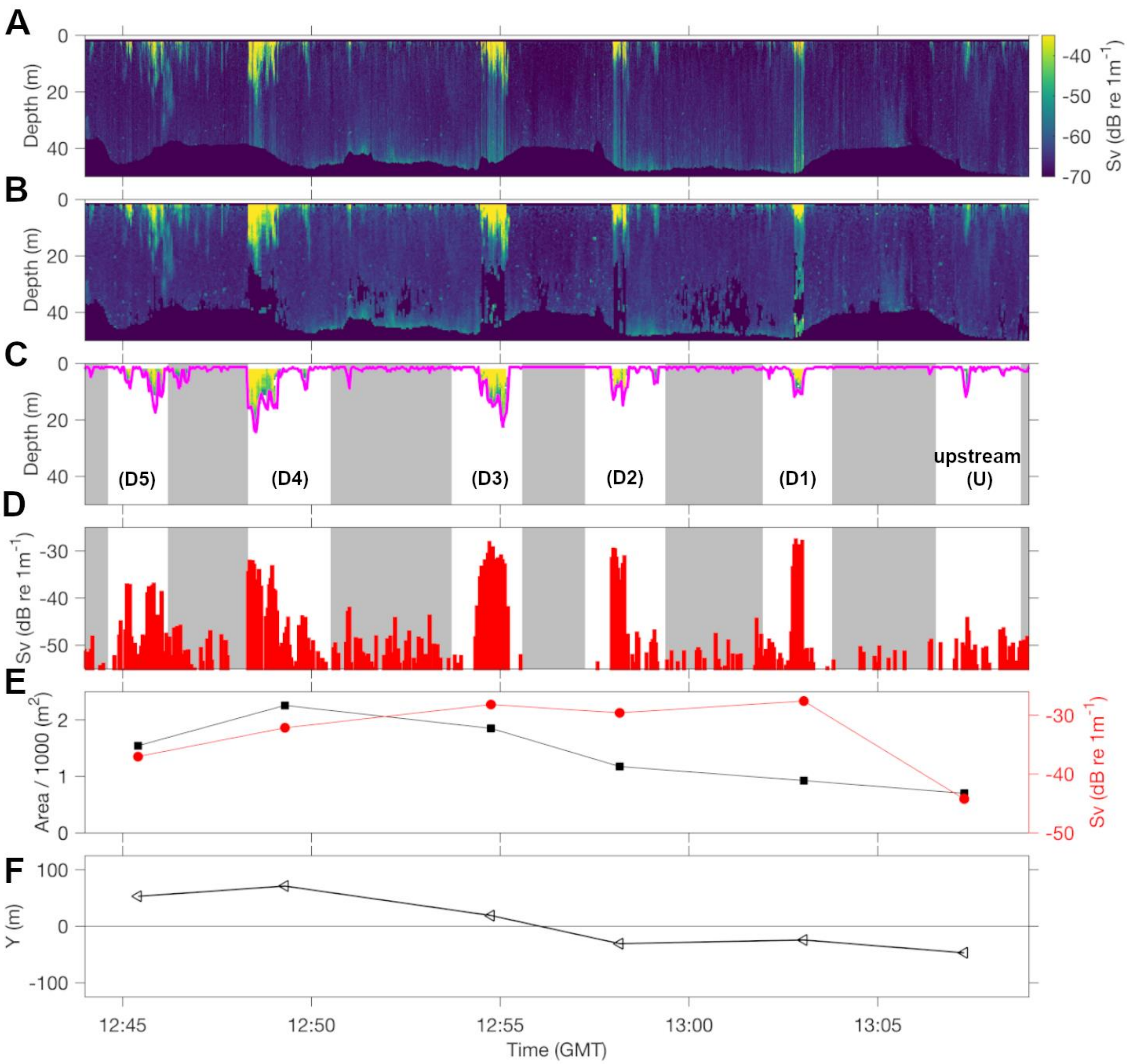

Fig. 5: Wake isolation and tracing using EK80 backscatter (Sv) from fine-scale transects. A Echogram of 200 kHz pre-processed data during ebb tide (17/04/2022, T5, mean ebb velocity= 2.8 ms^{-1}) showing mean volume backscattering strength (S_v; dB re $1m^{-1}$) as a function of depth (y axis) and time (x axis), with bad pings and seabed removed. The transect started downstream of the turbine (D5-D1) and ended with an upstream line (U), with the O2 being passed at about 13:05 (GMT). The colour bar represents the range of S_v values displayed in all echograms. **B** Denoising algorithm-processed echogram showing integrated mean volume backscatter strength data (gridded at a resolution of 5 pings along the track by 0.5 m depth bins). **C** Image-processed S_v data isolating and visualising surface-connected bubble entrainment by macro-turbulence using a -50 dB threshold. Highlighted in white are the cross-stream sections (Y=±100 m) of the transect in-line with the O2 platform. **D** Mean S_v within surface-connected bubble plume isolated in **C**. **E** Area (/1000; in m^2; left y-axis) and maximum backscattering strength (S_v; right y-axis) of bubble plume within each highlighted section. **F** Mean cross-stream (Y) location of the surface-connected bubble plume within each section, the O2 is located at Y=0.

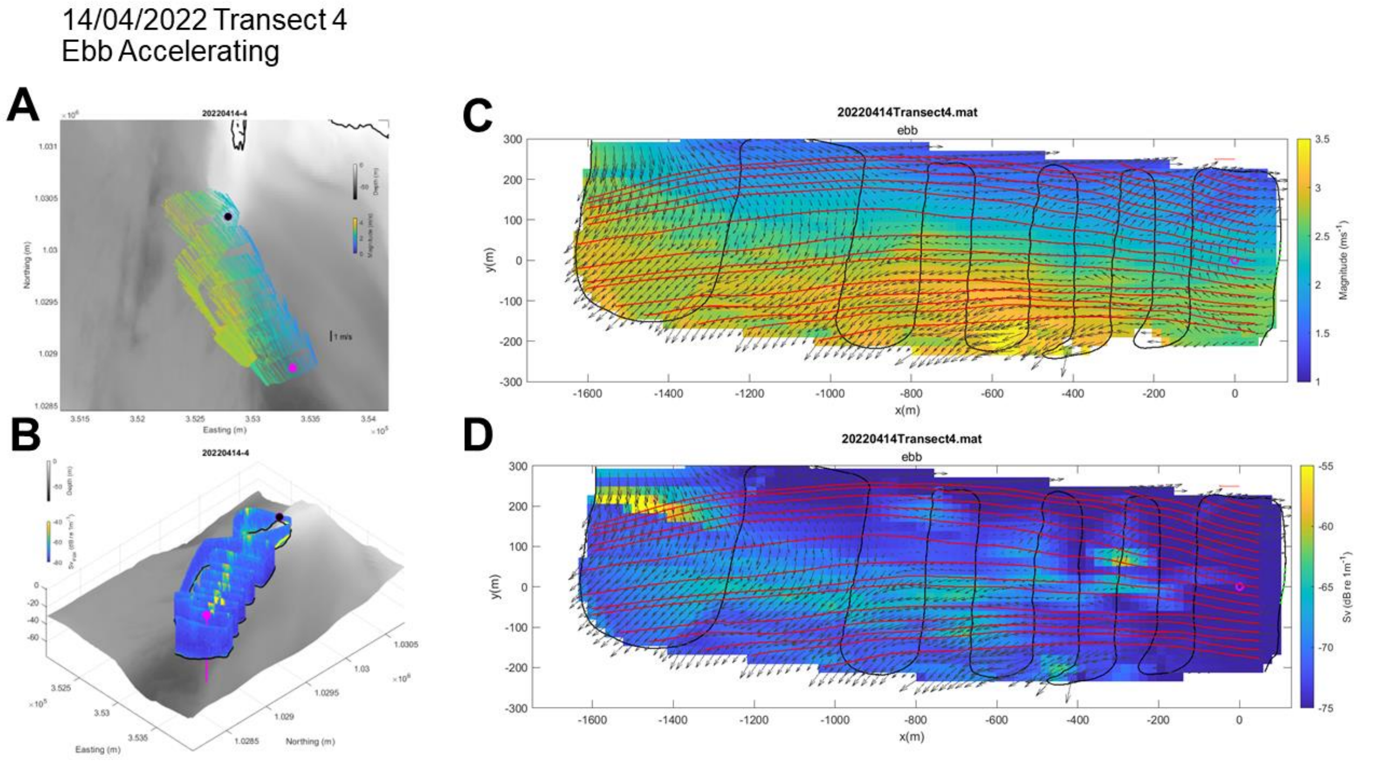

Fig. 6: Fine-scale transect extending 1600 m downstream of the O2 during ebb tidal flows. A Depth-averaged horizontal velocity with vectors coloured by magnitude (14/04/2022, T4, mean ebb velocity = 2.55 ms^{-1}). **B** Vertical distributions of ADCP-derived backscatter, a proxy for surface-connected bubble entrainment by macro-turbulence **C, D** Corresponding horizontal velocity magnitude and ADCP backscatter data rotated to the local coordinate system and gridded at 1D. Streamlines (red) show the behaviour of the flow cross- and downstream of the O2. For all plots, the mean location of the O2 tidal turbine is marked with a circle (magenta). Note, the strong scattering visible in the backscatter data in **D** (X=-1400-1600) is associated with the OpenHydro turbine installed in 2006 (marked with a black circle in A & B), consisting of two steel monopoles drilled into the seabed. The OpenHydro platform generated its own wake penetrating approximately 15 m deep over 30 m overall water depth. This is best visualised in **D** at x=-1300 to x= -1600 m. Boundary shape files: Boundary-Line™ shape files downloaded from EDINA Digimap Ordnance Survey Service. Bathymetry:©Crown Copyright/SeaZone Solutions Limited (2022). All Rights Reserved. Not to be used for navigation. Note, figures only provide an overview and the underlying ADCP-derived current flow data is available at DOI: https://doi.org/10.24382/244fae5d-2d16-4219-98f6-7aa96757ae49

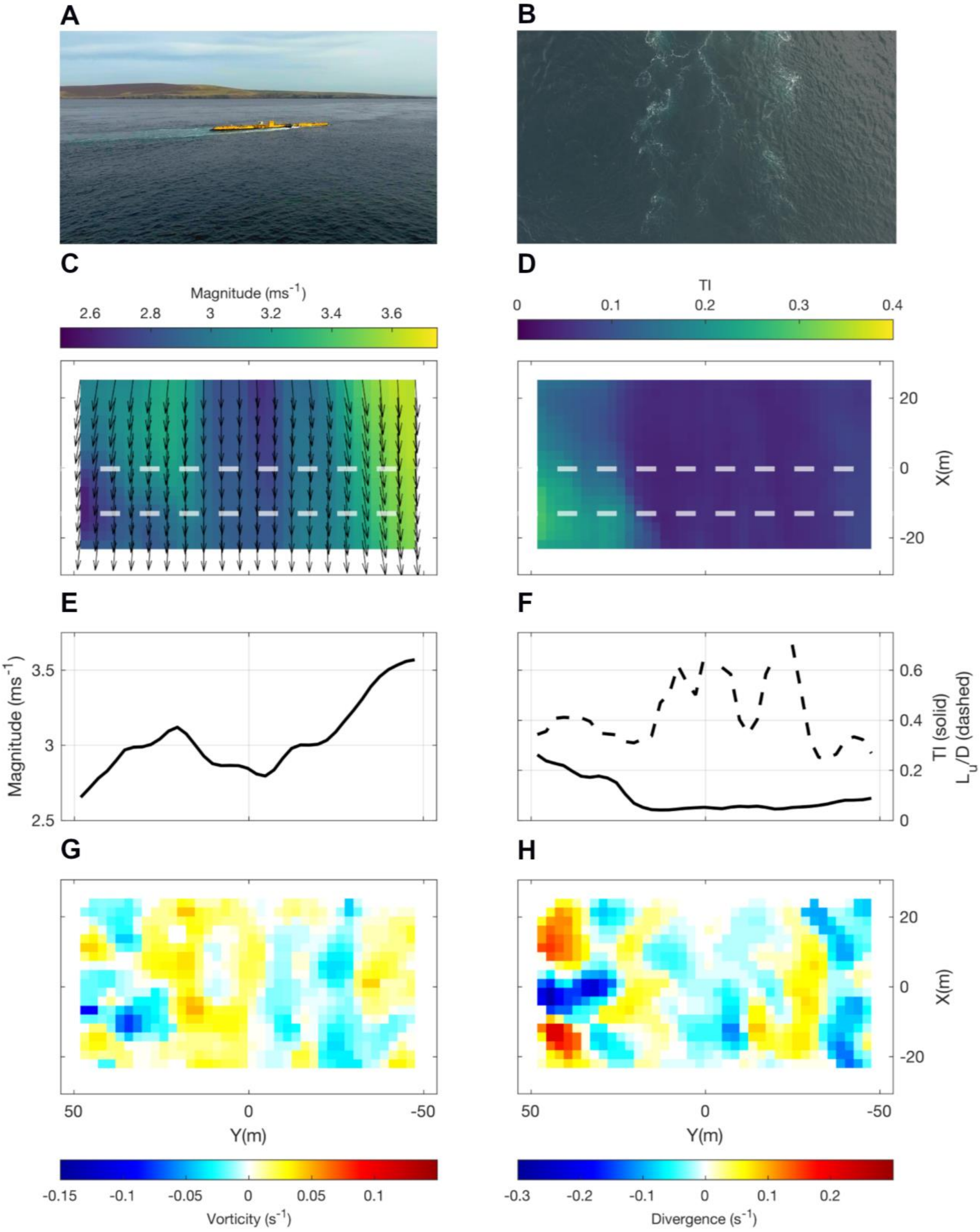

Fig. 7: PIV-derived surface current magnitude and turbulence across the O2 wake during ebb flow. A Oblique aerial drone image approaching the O2 platform during ebb flow (17/04/2022, mean ebb velocity = 3.18 ms^{-1}). **B** Aerial image of drone hover field of view (T6, hover 4, altitude=65 m) over the O2 wake (5.5D downstream) **C** Mean flow field coloured by horizontal velocity magnitude with velocity vectors overlaid and (**D**), turbulence intensity (TI), as calculated from the 2-min hover. **E** Spatial and temporal mean horizontal velocity magnitude and (**F**), turbulence intensity both calculated across the region bounded by dashed, white lines in **C** and **D**, highlighting the difference in horizontal velocity magnitude and TI across the wake area and adjacent to either side of the wake. The dashed lines in **F** are the turbulence length scales $L_u(\Delta X)$ normalised by the rotor diameter D. Regions of (**G**) vorticity (positive = anti-clockwise) and (**H**), divergence (positive = upwelling) and convergence (negative = downwelling) on either side of the O2 (frame=237, same as in B). The local coordinate system is centred around the mean O2 location.

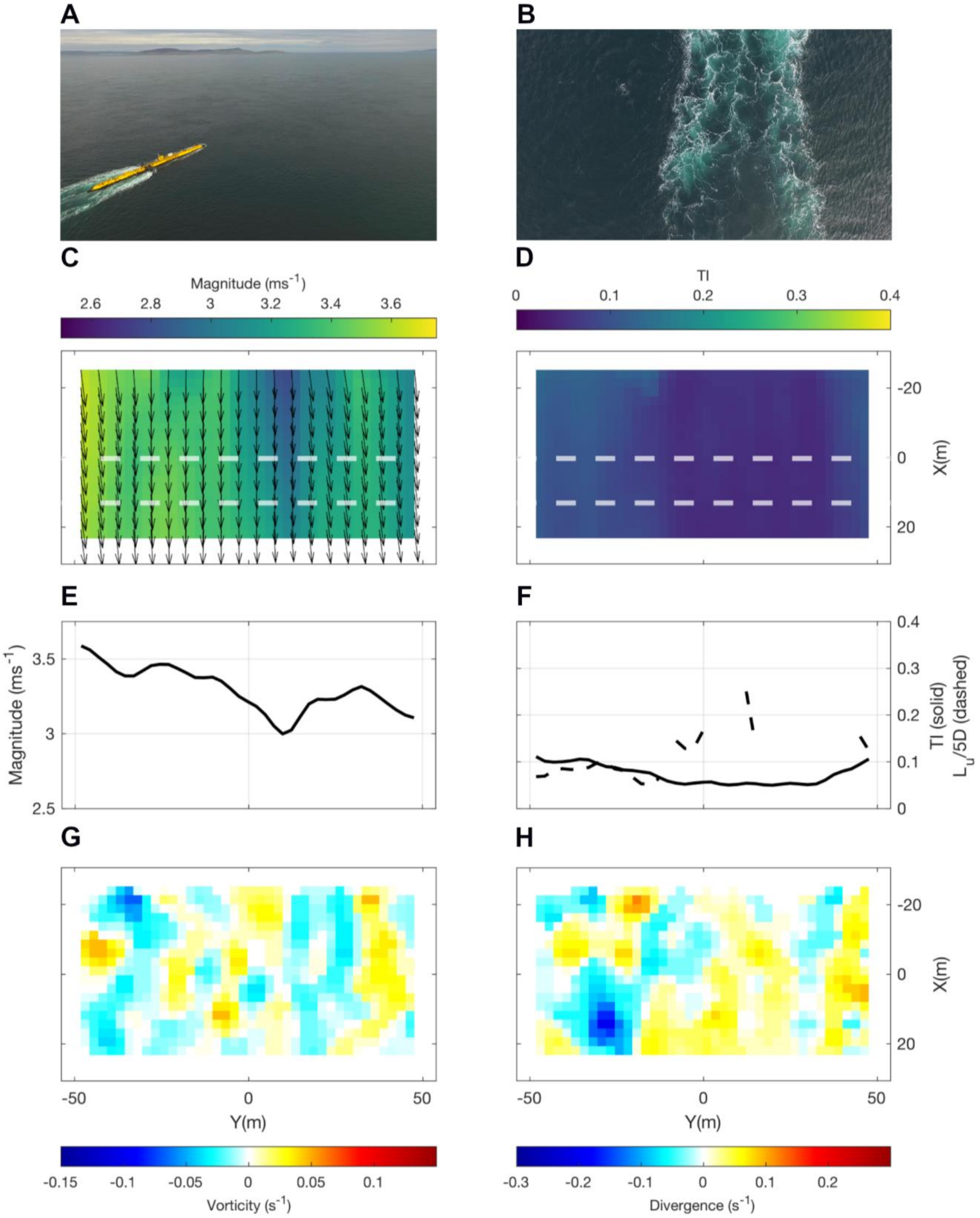

Fig. 8: PIV-derived surface current magnitude and turbulence across the O2 wake during flood flow. A Oblique aerial drone image approaching the O2 platform during flood flow (17/04/2022, mean flood velocity = 3.2 ms^{-1}). **B** Aerial image of drone hover field of view (T1, hover 4, altitude=65 m) over the O2 wake (5.5D downstream) **C** Mean flow field coloured by horizontal velocity magnitude with velocity vectors overlaid and (**D**), turbulence intensity (TI), as calculated from the 2-min hover. **E** Spatial and temporal mean horizontal velocity magnitude and (**F**), turbulence intensity both calculated across the region bounded by dashed, white lines in **C** and **D**, highlighting the difference in horizontal velocity magnitude and TI across the wake area and adjacent to either side of the wake. The dashed lines in **F** are the turbulence length scales $L_u(\Delta X)$ normalised by the rotor diameter D. Regions of (**G**) vorticity (positive = anti-clockwise) and (**H**), divergence (positive = upwelling) and convergence (negative = downwelling) on either side of the O2 (frame=237, same as in B). The local coordinate system is centred around the mean O2 location.

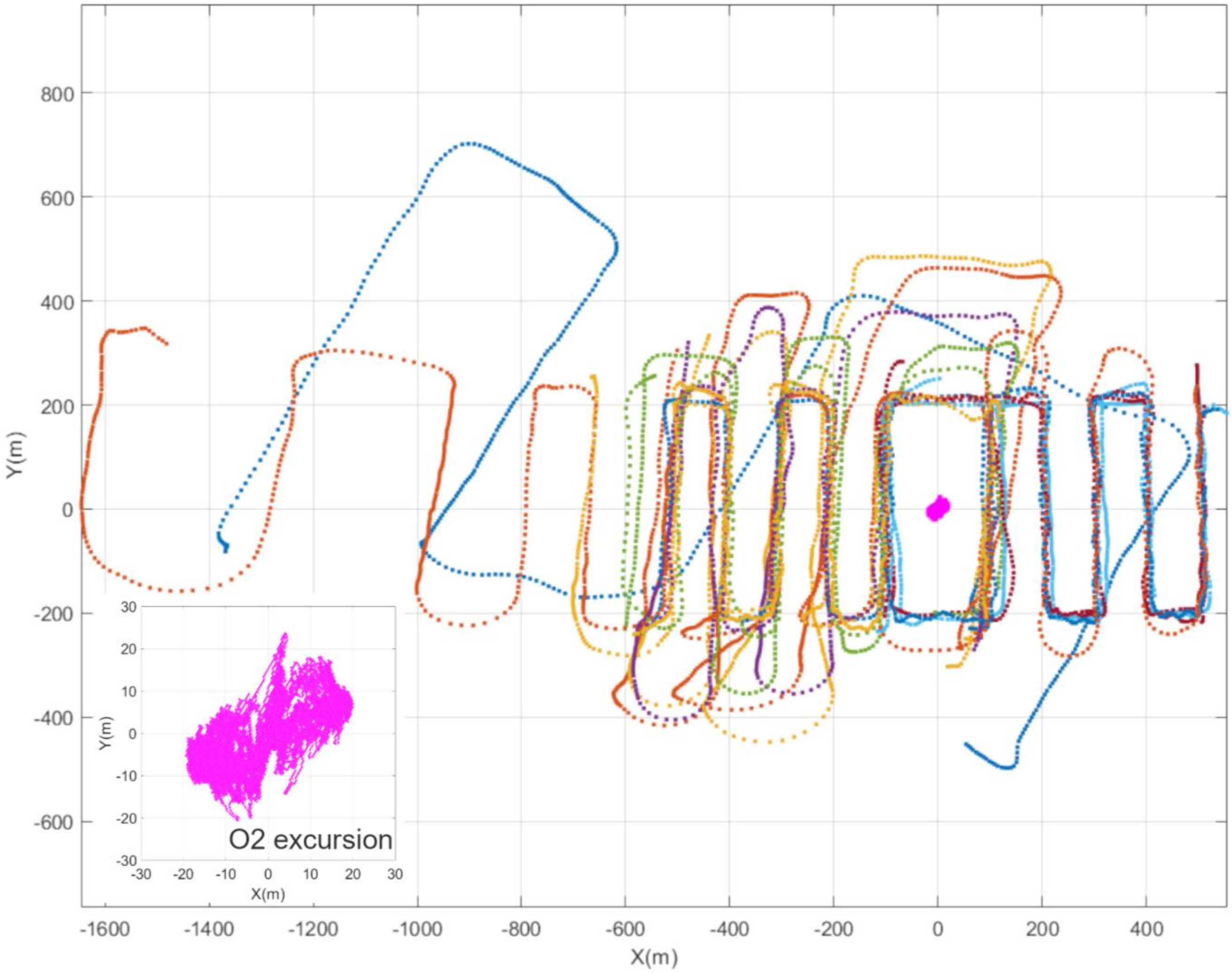

Fig. 9: Survey vessel transect lines during fine-scale surveys. Positions for all transect lines extracted from the ADCP data files (recorded at 4.22s intervals) showing the streamwise and cross-stream variations in surveys. Largest variation in transect line locations and orientation were during initial scoping transects on the 13th (dark blue points). Transect lines on the 14th and 17th that followed pre-defined waypoints are more tightly clustered at 100 m intervals upstream and downstream of the O2 location (magenta). The inset plot shows the overall excursion of the O2 platform during the entire sampling period. Note, the underlying data is available at DOI: https://doi.org/10.24382/244fae5d-2d16-4219-98f6-7aa96757ae49

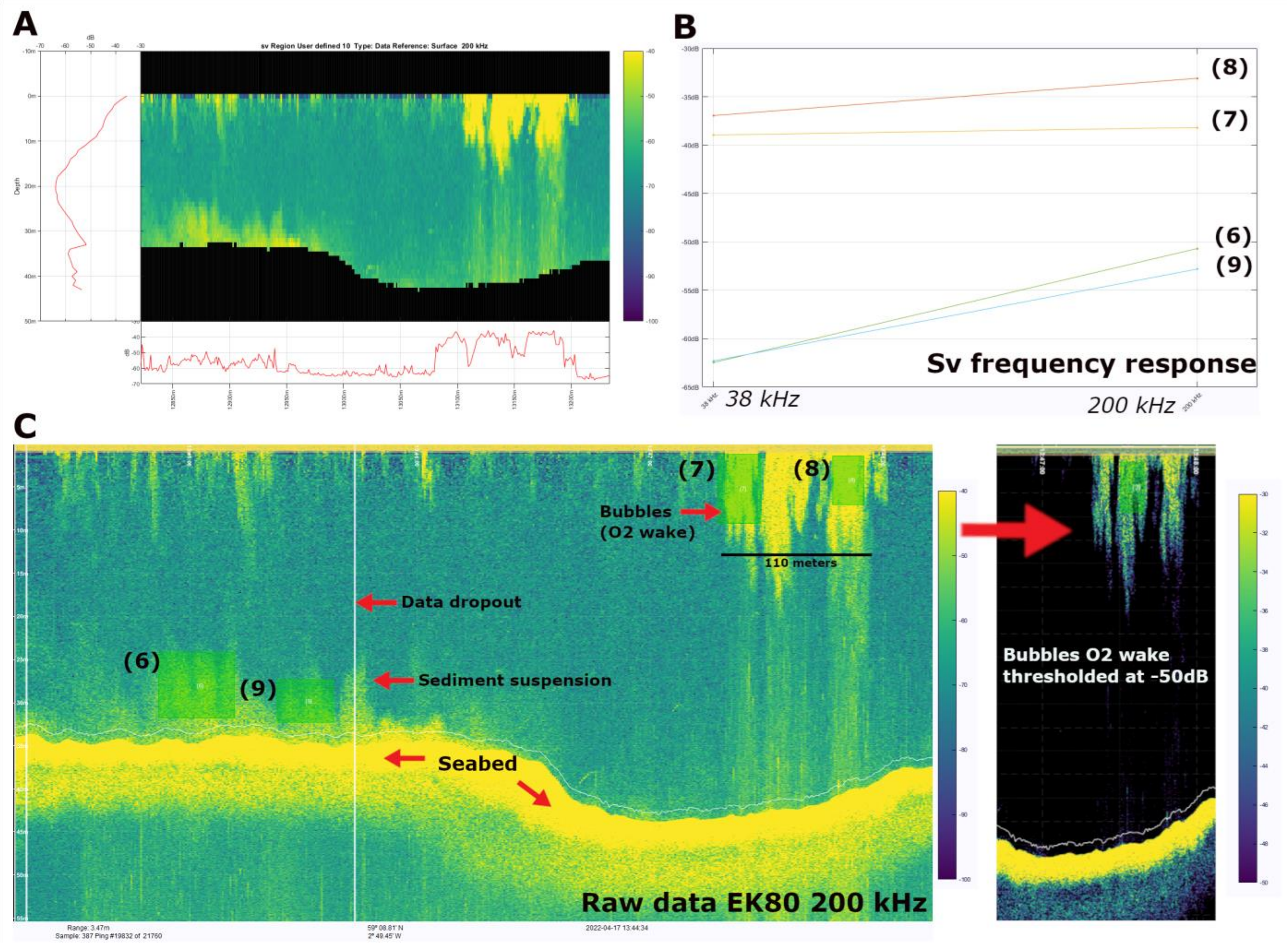

Fig. 10: Examples of backscattering sources and uncalibrated frequency response from bubbles and sediment. A Echo-integrated overview plot of region displayed in C. **B** Examples of volume backscattering measurements (S_v; dB re 1m^{-1}) from two sources displayed in C, bubbles (region 7 & 8) and sediment (region 6 & 9), as a function of the two available frequencies (38 & 200 kHz) shown on the x-axes. Note, although not calibrated, the increased scattering of the sediment regions at the 200 kHz frequency compared to the 38 kHz is consistent with studies using calibrated multi-channel, broadband echosounder systems. Overall, the bubbles scatter more strongly with >10 dB difference compared to the sediment. **C** An echogram (200 kHz) with labelled examples of backscattering sources or processes for part of an ebb tide transect (17/04/2022, T6, mean ebb velocity= 3.18 ms^{-1}) downstream of the O2 platform. The cross-stream extent of the bubbles associated with passing the O2 wake (time between 13:47 and 13:48 GMT) is approximately 110 m. The impacts of excess attenuation and multiple scattering are visible at the O2 wake below the bubble plume which appears to have a ‘‘tail’’ of echoes extending below the actual area of the wake, thus distorting the apparent shape and intensity of the wake. Multiple scattering can occur when the density of scatterers is high, and signals scattered from one particle are re-scattered from neighbouring particles (bouncing of echoes). In this case, the apparent wake seems to occupy the full water column, however de-noising and thresholding of the data to intensities consistent with adjacent portions of the wake was sufficient to isolate and trace the wake as per Supplementary Figure 5 and Figure 6 & 7 of the main manuscript. Small, unlabelled, and relatively intense backscatter signatures are most likely attributable to small fish or suspended biological material. Region 7 and 8 are dominated by backscatter from bubbles while region 6 and 9 correspond to scattering from suspended sediment.

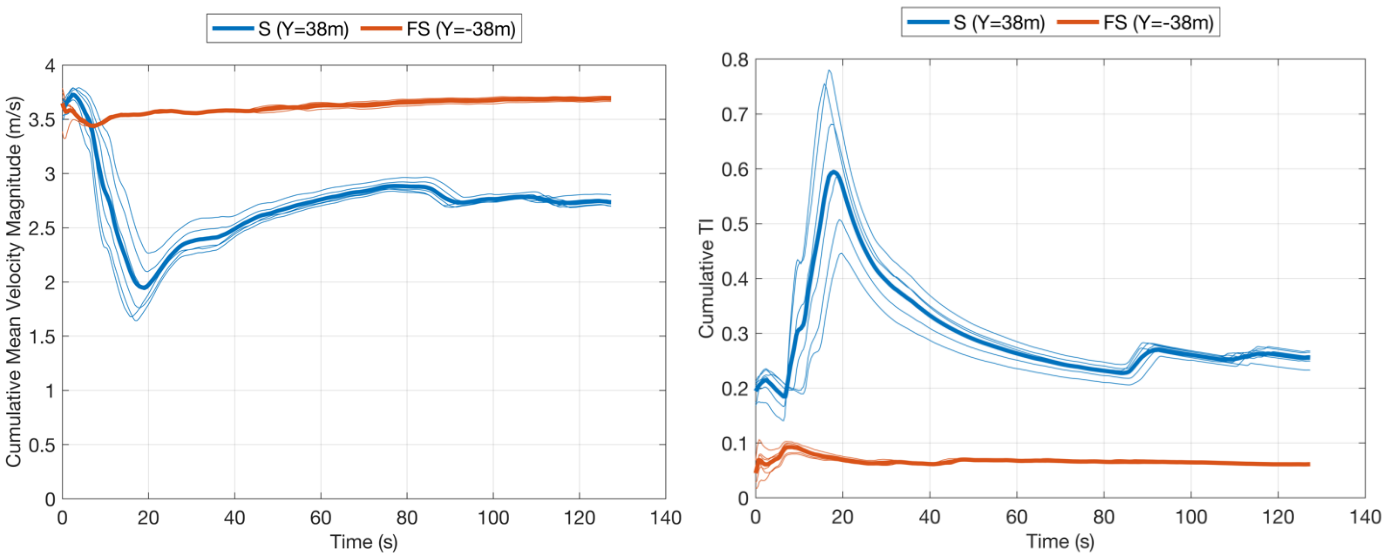

Fig. 11: Convergence of flow parameters derived from PIV data. Cumulative mean over time of the velocity magnitude (left) and cumulative turbulence intensity (right), both spatially-averaged in the alongstream-direction (thick lines) between the white dashed lines on Figure S3C, from two different individual Y locations showing good convergence of the values in the freestream (FS, Y= -38 m), and less good convergence in the shear line (S, Y= 38 m), by which time the total number of samples incorporated is 2,868. The individual data series that comprise the spatial average are shown by the associated thin lines, comprising a maximum of 478 samples each.

Supplementary Methods 1: Large-scale Particle Image Velocimetry (LSPIV)

Lab-based particle image velocimetry (PIV) relies on seeding particles for flow visualization and velocity measurements in controlled settings. In contrast, 'Large-scale particle image velocimetry (LSPIV)' is commonly referred to when applied in natural environments such as rivers, estuaries, or oceans. LSPIV utilizes natural features or tracers for image-based or remote sensing-based velocity measurements, making it well-suited for studying large-scale flows (on the order of meters) in natural water bodies. Typically [Supp1-5], LSPIV doesn't require seeding the flow with particles, instead relying on natural features or tracers present in the fluid, such as surface waves, boils, debris, foam lines [Supp6], or other high-contrast features. Unlike controlled environments such as laboratories or flumes where flows are seeded with particles, LSPIV operates in unconstrained natural settings.

Although there have been attempts to seed more constrained natural river flows with particles[Supp7], practical considerations, such as the quantity of seeds needed and the non-constrained channel flow of tidal stream sites, alongside ecological concerns, generally make this approach unfeasible. Given the ecological sensitivity of sites like tidal streams, with diverse marine fauna populations, the potential biological and ecological effects of particle releases remain unknown and are thus deemed impractical for such natural environments. Other examples of using naturally occurring tracers for PIV analysis include using natural suspended particles (sediment, plankton) and submersible PIV[Supp8] to quantify turbulence in the coastal environment, and using PIV on snowflakes to study the wake of wind turbines[Supp9].

LSPIV has proven to be a valid and cost-effective image-based tool for measuring surface velocity using natural tracers in various fluvial systems, particularly in field applications. The PIV methodology relies on the cross-correlation between small regions (or “windows”) of successive images. Sufficient natural tracer features within each window are necessary to obtain good quality data, determined by the correlation threshold. Smooth water yields very low correlation values, whereas textured water yields higher correlation values. Spurious artefacts, such as sun glint, would also introduce errors that a correlation threshold alone will not remove.

We have developed and applied our own custom LSPIV analysis code to extract water surface velocity fields (speed and direction of the flow) every 0.266 s (8 frames) from each drone video sequence. We use the following simple

steps to ensure robustness and reliability of the resulting velocity vector fields, without using the more advanced methods that are commonly used in laboratory-based PIV where particle seeding density and lighting are controlled to give optimal conditions. At each 0.266 s interval, four consecutive video frames (recorded at 30 fps, representing 0.1 s total duration) were used. The green colour channel (selected as most representative of the water colour) of each frame were extracted and then corrected for camera lens distortion by applying a transformation matrix prepared using the MATLAB camera calibration toolbox and a standard chequer-board technique. A simple cross-correlation algorithm[Supp10] between consecutive frames was then applied using 65 × 65 pixel windows with 50% overlap and 128-pixel clear border. This includes sub-pixel localization of the maximum correlation peak using a two-dimensional quadratic function fitted through the 3x3 pixel neighbourhood. This results in fields of 20 × 39 velocity vectors extracted per frame-pair with a correlation coefficient reported for each vector indicating its quality. A minimum correlation threshold of 0.6 is applied, however for all data series reported here the minimum correlation value obtained was about 0.8. Randomly selected examples of the cross-correlation distribution in the vicinity of individual vector locations are shown in Fig. S12. Predominantly the distributions are unimodal, making a simple correlation threshold quality metric more suitable than metrics derived from multi-modal distributions (e.g. signal-to-noise ratio). To reduce spurious effects from sun glint, a 3 × 3 × 3 median filter (filtering in both spatial dimensions and time) was applied across the three vector fields extracted from the four consecutive video frames providing one clean velocity field every 0.25 s through the video sequence that were then scaled according to the drone's altitude. Each 2-min hover results in about 500 clean velocity fields. As shown by the continuous distributions of pixel displacements in Fig. S13, the combination of selected window size, pixel resolution and relative scale of natural features delivers data unaffected by "pixel locking" artifacts (strong bias to integer pixel displacements) that can impact PIV measurements using poorly resolved seeded tracer particles.

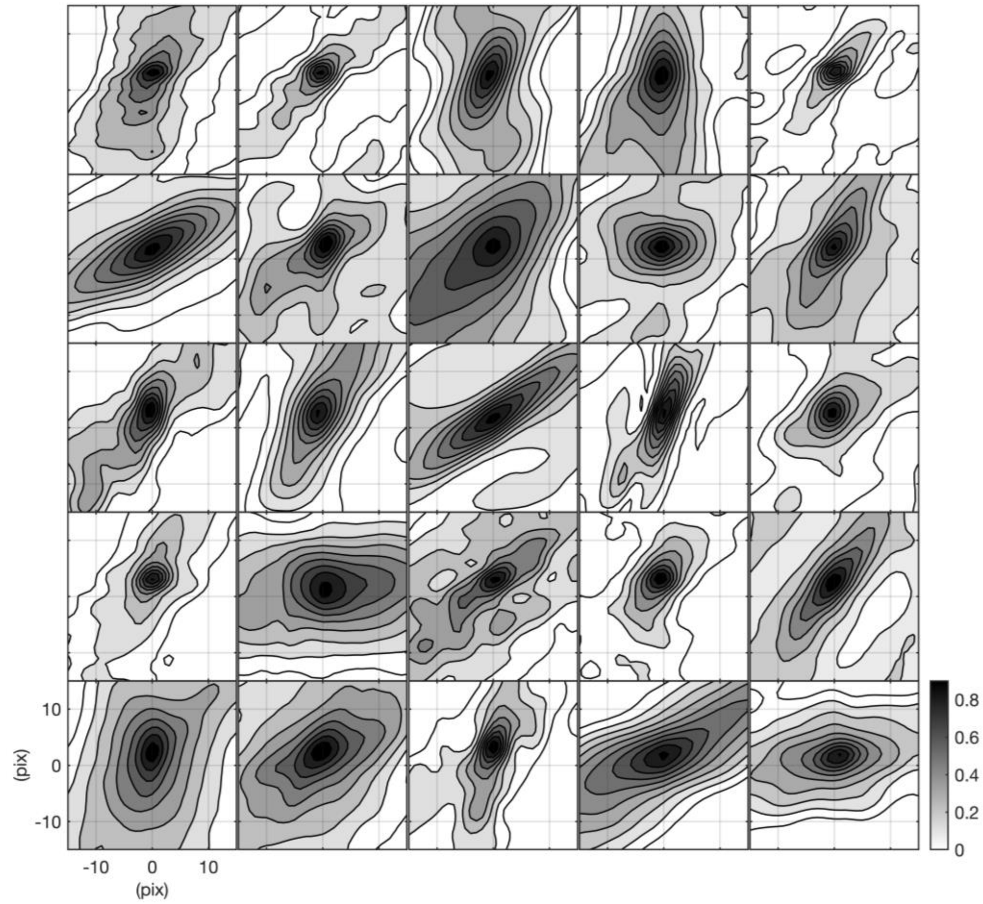

Fig. 12: Spatial (pixel space) distributions of cross-correlation at 25 randomly selected instantaneous vector locations upstream of the O2. The determination of the cross-correlation peak location provides the inter-frame displacement and hence velocity. Filled contours are at correlation intervals of 0.1 with values given by the colour bar. Ebb flow transects on 17/04/2022 (T6, hover 1, altitude=65 m).

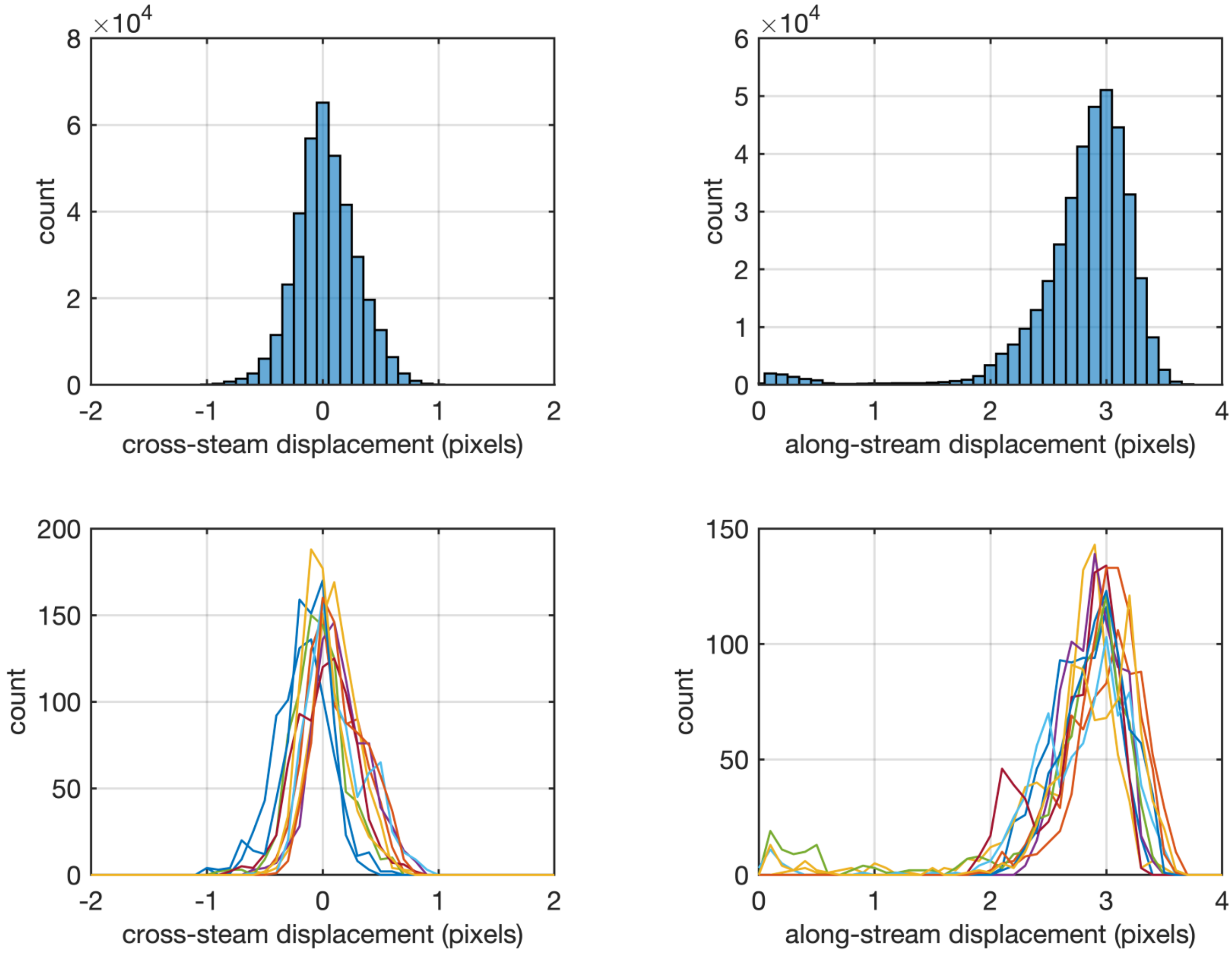

Fig. 13: Histograms of measured cross-correlation peak displacement to assess peak-locking. Histograms of LSPIV-derived displacement data (pixels) from which velocity vectors are determined upstream of the O2 (17/04/2022, T6, hover 1). The upper panels show all vector locations and time intervals for an entire 2-min hover, while the lower panels show all vector locations at 10 random time instants within the same hover.

Supplementary Methods 2: Turbulence length scales

We have explored two possible methods to estimate the turbulence length scale (L_u) from the PIV data. Firstly, following[Supp11], we apply Taylor's frozen field to individual vector locations, calculating the autocorrelation function ($R_{u,x}(\overline{U}\Delta t)$) and then applying alongstream spatial averaging prior to integration up to the $1/e$ crossing (Figure S14 (left) and equation 8 in [Supp11]):

$$L_{u,x} = \overline{U} \int_0 R_{u,x}(t) d\Delta t$$

Secondly, we use the spatial nature of the PIV data to estimate L_u by calculating the mean correlation as a function of streamwise separation ($R_{u,x}(\Delta X)$) prior to integration up to the separation needed for the correlation curve to decrease to $1/e$ (Figure S14 (right)). The $1/e$ threshold has been previously used[Supp11] in cases where the correlation function does not cross the horizontal axis due to random velocity fluctuations. Under some circumstances, the correlation still does not drop below this threshold, in which case it is not possible to return a value for L_u.

As seen in Figure S15, the two different methods for estimating L_u provide some differences in results. During the ebb flow at the inflow location (Fig. S15 left) the two estimates are generally in good agreement in the cross-stream extent, with elevated values closer to the Eday shear line (positive Y) directly corresponding to later crossing of the $1/e$ threshold for the shear-line curves in Figure S14. However, within the wake (Fig. S15 right, Y = -25 to 25 m) there

are large differences, with elevated L_u values using the spatial method and maybe even a decrease in turbulence length scale when calculated using Taylor's frozen field. This indicates that at a single cross-stream location within the wake, the nature of the turbulence leads to rapid temporal decorrelation but has higher alongstream spatial correlation.

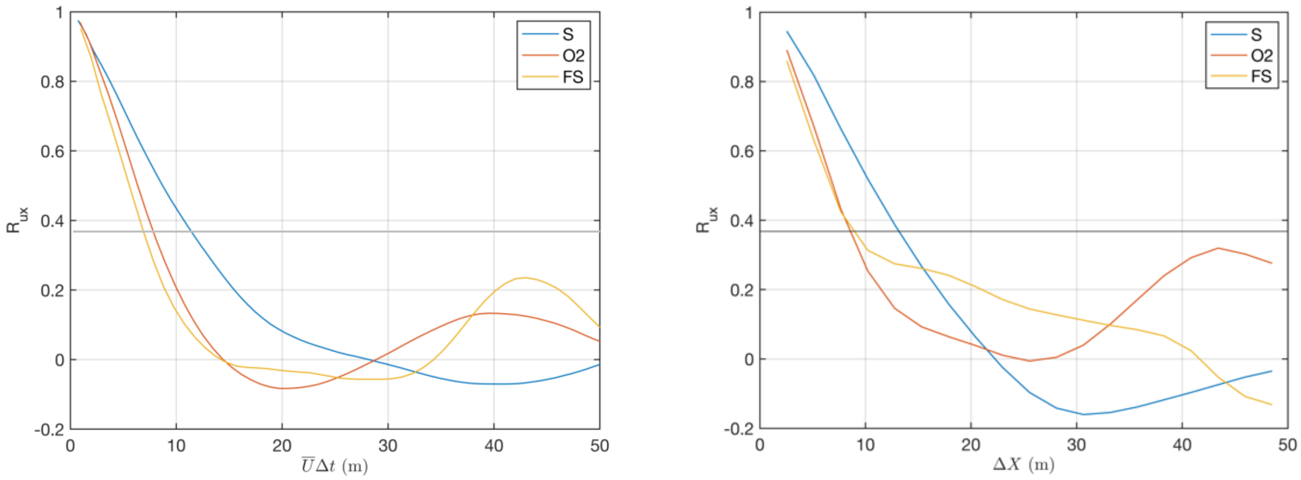

Fig. 14: Estimation of turbulence length scale (Lu). (left) alongstream spatially-averaged autocorrelation as a function of streamwise distance determined using Taylor's frozen field ($R_{u,x}(\overline{U}\Delta t)$). (right) mean correlation as a function of streamwise separation ($R_{u,x}(\Delta X)$). In both, the horizontal line demarcates the $1/e$ threshold. S, O2 and FS indicate shear-line, O2 and free stream cross-stream locations, respectively. Ebb flow transects on 17/04/2022 (T6, hover 1, altitude=65 m).

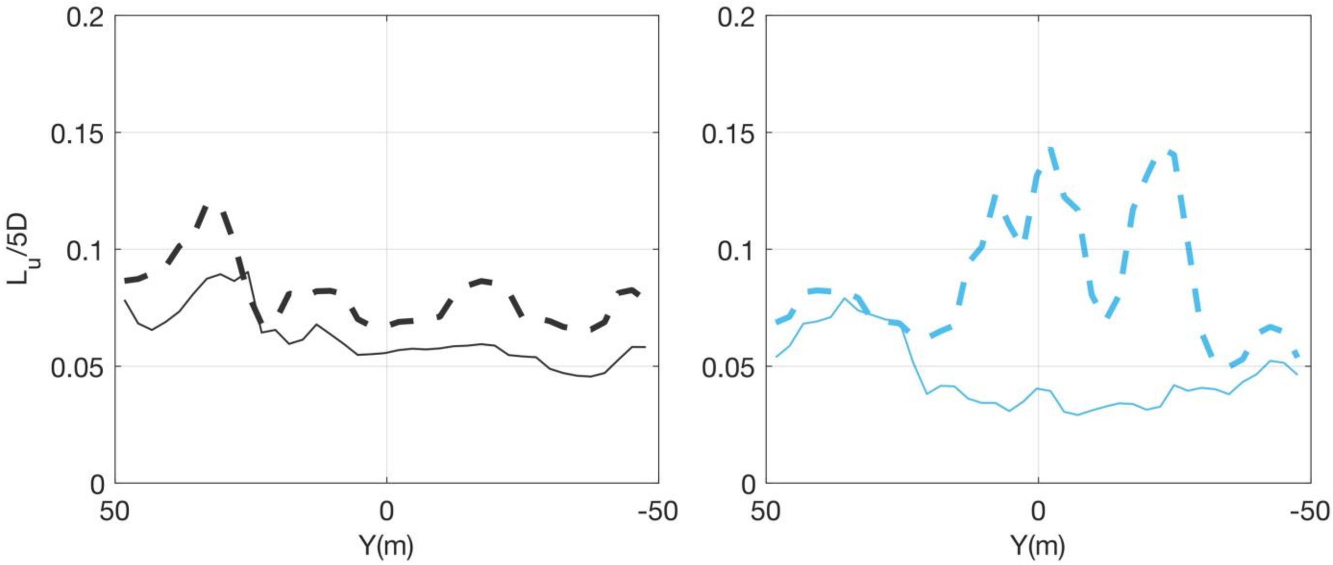

Fig. 15: Comparison of the two methods for estimation of turbulence length scale (Lu). Ebb flow hovers on 17/04/2022 (T6, altitude=65 m) showing the (left) inflow, hover 1, and (right) 5.5D, hover 4, wake area. In each, thin solid lines are $L_u(\overline{U}\Delta t)$ and dashed lines are $L_u(\Delta X)$.

Supplementary Methods 3: Assessment of Drone Stability and impact on turbulence parameters

In order to assess the stability of the drone during each sampling hover used for LSPIV analysis, we have followed the procedure of [Supp12]. Briefly, the drone utilised during the surveys was re-flown over a static, textured scene (cobble beach). The in-flight wind conditions, extracted from the Airdata UAV log management system that applies a drone-model specific aerodynamic model to the flight metrics, during this test flight are compared to those encountered during the surveys in Table S2. Importantly, we report both the mean and standard deviation of the wind speed derived from the UAV logs at 5 s intervals throughout the hovers. It can be seen that the test flight conditions were more extreme (in both mean speed and variation, or gusts) than encountered during the surveys. The horizontal and vertical stability of the drone is indicated by the standard deviation of the position and altitude as well as their maximum horizontal and vertical displacements during the hover (see Table S2). The yaw stability of the drone is indicated by the standard deviation in heading. Throughout the surveys when the gusts were weak (Std of wind<0.4 m/s), the variations in drone position, altitude and yaw are much smaller than under the strong gusts (Std of wind>2.5 m/s) of the subsequent test flight, highlighting the importance of including gusts in these drone stability assessments.

The recorded video of the static scene was analysed using our LSPIV method detailed above to provide a direct measure of the velocity contamination from drone movement under these extreme test conditions. The resulting root mean square (RMS) velocity fluctuation within the central section of the field of view is 0.149 ms^{-1}. This increases to 0.195 ms^{-1} at the peripheries of the sample area where the combined effects of vertical and rotational movements are most pronounced. Therefore, a reasonable and conservative sensitivity threshold can be determined, for example for Turbulence Intensity, by comparing the RMS velocity fluctuation value obtained under these extreme test conditions to the typical mean flow speeds encountered during the surveys (of order 2ms^{-1}), resulting in a TI sensitivity threshold of 0.149 ms^{-1}/ 2 ms^{-1} = 0.075.

Table 2: Drone stability parameters for hover segments and test flight over beach scene.

Figure Ref	Date Time Transect Hover	Duration (s)	Mean Wind (m/s)	Std Wind (m/s)	Std Horizontal Displacement (m)	Maximum Horizontal Displacement (m)	Min/Mean/Max Altitude (m)	Std Altitude (m)	Std Heading (°)
5	17/04/2022 13:31:25 T6 h2	124	7.88	0.18	0.06	0.33	64.40/64.65/64.80	0.09	0.07
S1	17/04/2022 13:28:58 T6 h1	128	8.29	0.39	0.04	0.19	64.60/64.81/65.20	0.10	0.21
S2	17/04/2022 07:56:16 T1 h2	129	2.96	0.38	0.05	0.32	64.70/64.92/65.10	0.08	0.17
S3	17/04/2022 13:36:04 T6 h4	126	8.14	0.28	0.04	0.28	64.50/64.75/64.90	0.10	0.09
S4	17/04/2022 08:00:56 T1 h4	128	4.50	0.17	0.05	0.32	64.50/64.78/65.00	0.09	0.16
**Test**	**15/03/2024 14:47:54**	**181**	**9.70**	**2.54**	**0.17**	**0.81**	**64.50/65.05/65.50**	**0.20**	**0.48**

Supplementary References

1. Muste, M., I. Fujita, and A. Hauet (2008), Large-scale particle image velocimetry for measurements in riverine environments, Water Resour. Res., 44, W00D19, https://doi.org/10.1029/2008WR006950
2. Lewis, Q. W., and B. L. Rhoads (2015), Resolving two-dimensional flow structure in rivers using large-scale particle image velocimetry: An example from a stream confluence, Water Resour. Res., 51, 7977–7994, https://doi.org/10.1002/2015WR017783
3. Tauro, F., Petroselli, A., Arcangeletti, E., (2016). Assessment of drone-based surface flow observations. Hydrol. Process. 30 (7), 1114–1130. https://doi.org/10.1002/hyp.10698
4. Tong Jin, Qian Liao (2019) Application of large scale PIV in river surface turbulence measurements and water depth estimation. Flow Measurement and Instrumentation. https://doi.org/10.1016/j.flowmeasinst.2019.03.001.
5. Zhu, X., & Lipeme Kouyi, G. (2019). An analysis of LSPIV-based surface velocity measurement techniques for stormwater detention basin management. Water Resources Research, 55, 888–903. https://doi.org/10.1029/2018WR023813
6. Lieber, L., Füchtencordsjürgen, C., Hilder, R.L., Revering, P.J., Siekmann, I., Langrock, R. and Nimmo-Smith, W.A.M. (2022), Selective foraging behavior of seabirds in small-scale slicks. Limnol. Oceanogr. Lett, 8: 286-294. https://doi.org/10.1002/lol2.10289
7. Strelnikova D, Paulus G, Käfer S, Anders K-H, Mayr P, Mader H, Scherling U, Schneeberger R. (2020) Drone-Based Optical Measurements of Heterogeneous Surface Velocity Fields around Fish Passages at Hydropower Dams. Remote Sensing. 12(3):384. https://doi.org/10.3390/rs12030384
8. Nimmo-Smith, W. A. M., J. Katz, and T. R. Osborn, 2005: On the Structure of Turbulence in the Bottom Boundary Layer of the Coastal Ocean. J. Phys. Oceanogr., 35, 72–93, https://doi.org/10.1175/JPO-2673.1.
9. Hong, J., Toloui, M., Chamorro, L. P., Guala, M., Howard, K., Riley, S., Tucker, J., & Sotiropoulos, F. (2014). Natural snowfall reveals large-scale flow structures in the wake of a 2.5-MW wind turbine. *Nature Communications*, 5(May). https://doi.org/10.1038/ncomms5216
10. Raffel M, Willert CE, Wereley ST, Kompenhans J. 2007 Particle image velocimetry: a practical guide. New York: Springer Press. 79–92
11. Trush, A., Pospíšil, S. & Kozmar, H. Comparison of turbulence integral length scale determination methods. in WIT Transactions on Engineering Sciences vol. 128 113–123 (WITPress, 2020).
12. Fairley, I. et al. Drone-based large-scale particle image velocimetry applied to tidal stream energy resource assessment. Renew Energy 196, 839–855 (2022)
